# Covalent Organic Frameworks‐Delivered Reuterin Drives Trained Immunity in Tumor‐Associated Macrophages to Enhance Melanoma Immunotherapy via Glycerophospholipid Metabolism

**DOI:** 10.1002/advs.202504784

**Published:** 2025-06-30

**Authors:** Jian‐Gang Zhang, Xiao‐Mei Zhang, Xi Wu, Cheng‐Kai Zhou, Zhen‐Zhen Liu, Xue‐Yue Luo, Liang Zhang, Wei Chen, Yong‐Jun Yang

**Affiliations:** ^1^ College of Veterinary Medicine Jilin University Changchun Jilin Province 130062 P. R. China

**Keywords:** intratumor bacteria, *Limosilactobacillus reuteri*, melanoma, reuterin, trained immunity, tumor‐associated macrophages

## Abstract

The gut microbiota is increasingly recognized as a promising therapeutic target in cancer treatment. However, the specific mechanisms by which gut bacteria and their metabolites exert therapeutic effects in melanoma remain poorly understood. In this study, it is unexpectedly demonstrated that prophylactic supplementation with *Limosilactobacillus reuteri* exhibits significant tumor‐suppressive properties, primarily mediated by its secreted metabolite, reuterin. This metabolite induces trained immunity through macrophage metabolic reprogramming, thereby enhancing antitumor immune responses. Mechanistically, this process involves stabilizing HIF‐1α via the AHR‐ROS signaling pathway, enhancing glycerophospholipid metabolism, and elevating arachidonic acid levels, thereby amplifying the trained immunity response. Similar to reuterin, arachidonic acid also induces trained immunity and facilitates macrophage‐mediated tumor cell killing. To enhance its therapeutic efficacy, reuterin is encapsulated in covalent organic frameworks (COFs). COF‐Reuterin demonstrates superior effects in tumor‐associated macrophages (TAMs), remodulating intratumor bacteria and directly facilitating tumor cell killing. Notably, COF‐Reuterin demonstrates superior therapeutic efficacy compared to cisplatin. Furthermore, COF‐Reuterin reprogrammed TAMs from an M2 to an M1 phenotype, increasing CD8^+^ T cell infiltration and decreasing myeloid‐derived suppressor cells (MDSCs), reshaping the immunosuppressive tumor microenvironment. These findings highlight the potential of probiotics and their metabolites in the metabolic reprogramming of TAMs, offering a promising cancer therapeutic approach.

## Graphical abstract

Covalent organic framework (COF)‐reuterin induces trained immunity in tumor‐associated macrophages, promoting the generation of nitric oxide and reactive oxygen species, thereby enhancing the antitumor immune response. Additionally, COF‐Reuterin directly kills tumor cells while simultaneously eradicating pathogenic intratumoural bacteria. Thus, COF‐Reuterin enhances both innate and adaptive antitumor immunity, inhibiting tumor growth and remodeling the tumor microenvironment.

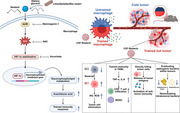

## 1 Introduction

Melanoma, the deadliest form of skin cancer, originates from melanocytes and is driven by complex molecular mechanisms. Its global incidence continues to rise, placing increasing strain on healthcare systems. In certain regions, the incidence of melanoma increases by ≈3% annually, and it accounts for nearly 90% of skin cancer‐related deaths.^[^ ^1^ ^]^ In 2023, the United States reported 97610 new cases of melanoma, resulting in an estimated 7990 deaths.^[^ ^2^ ^]^ Despite advances in targeted therapies and immunotherapy, the 5‐year survival rate for advanced melanoma has improved from less than 10% to ≈30%,^[^ ^3^ ^]^ but challenges such as resistance to these therapies and the variable efficacy of immunotherapy remain. These ongoing challenges underscore the complexity of melanoma treatment and emphasize the urgent need for innovative approaches. Personalized cancer therapies that enhance the immune response to melanoma and address current treatment limitations are becoming increasingly critical.

Trained immunity refers to a process in which innate immune cells undergo metabolic and epigenetic reprogramming following an initial stimulus, resulting in enhanced nonspecific immune responses upon re‐exposure to either homologous or heterologous challenges.^[^ ^4^ ^]^ Inducing trained immunity in innate immune cells presents a promising strategy for cancer management, primarily by augmenting antitumor immune responses.^[^ ^5^ ^]^ Research has shown that inducing trained immunity in hematopoietic progenitor cells can effectively inhibit lung cancer growth.^[^ ^6^ ^]^ Furthermore, activating trained immunity in alveolar macrophages, which reside in mucosal tissues, has been demonstrated to suppress melanoma lung metastasis in mice,^[^ ^7^ ^]^ highlighting the role of trained immunity in lung macrophages in combating metastatic tumor cells. Emerging evidence further suggests that tumor cells can reactivate these trained immune cells,^[^ ^8^ ^]^ emphasizing their potential in cancer immunotherapy. However, limited research exists on whether trained immunity can be effectively induced in tumor‐associated macrophages (TAMs) within the tumor microenvironment (TME) to stimulate antitumor immune responses. Current cancer therapies primarily focus on promoting the polarization of M1‐TAMs or reprogramming M2‐TAMs into an M1‐like antitumoral phenotype.^[^ ^9^ ^]^ This underscores the urgent need for innovative strategies that harness and regulate trained immunity in TAMs to enhance antitumor responses, paving the way for novel therapeutic approaches.

Gut microbiota can influence cancer progression and the outcomes of various cancer therapies, including chemotherapy, immunotherapy, and radiotherapy. As such, strategies aimed at modulating gut microbiota hold promise for enhancing cancer prevention and treatment.^[^ ^10^ ^]^ Growing understanding has led to an increasing demand among cancer patients actively seeking probiotics to improve health and potentially support cancer treatment.^[^ ^11^ ^]^ Specific bacterial species have been linked to both tumor promotion and suppression in colorectal cancer. For instance, *Fusobacterium nucleatum*, *Bacteroides fragilis*, and *Streptococcus gallolyticus* have been implicated in promoting tumorigenesis, whereas probiotics such as *Clostridium butyricum*, *Streptococcus thermophilus*, *Lacticaseibacillus paracasei*, and *Ruminococcus gnavus* have demonstrated antitumor effects.^[^ ^12^ ^]^ However, the precise mechanisms through which probiotics and their metabolites influence cancer progression, particularly by modulating innate immune memory, remain poorly understood. This critical knowledge gap restricts the full potential of probiotics as a viable therapeutic strategy in cancer immunotherapy.

Here, we demonstrate that the metabolite reuterin, produced by *Limosilactobacillus reuteri*, induces trained immunity in TAMs, leading to potent antimelanoma effects. Specifically, pretreatment with reuterin triggers trained immunity in macrophages, enhancing macrophage cytotoxicity, nitric oxide (NO) production, and reactive oxygen species (ROS) generation, thereby significantly inhibiting tumor growth. The antitumor effects of reuterin‐induced trained immunity are attributed to metabolic reprogramming in macrophages, driving TAMs to acquire an antitumor phenotype. To maximize the therapeutic efficacy of reuterin, efficient drug delivery is critical. Recent studies have underscored the potential of covalent organic frameworks (COFs) as a versatile drug delivery system for cancer therapy.^[^ ^13^ ^]^ COFs, with their high surface area, tunable porosity, and functionalized surface chemistry, serve as effective carriers for encapsulating and targeting therapeutic agents.^[^ ^14^ ^]^ Consequently, reuterin was incorporated into the COFs, which not only facilitated the induction of trained immunity in TAMs but also modulated the intratumor bacteria and enhanced tumor cell killing. This strategy significantly suppressed tumor progression, demonstrating the potential of reuterin‐loaded COFs as a promising immunotherapeutic approach for melanoma.

## 2 Results

### 2.1 Prophylactic Supplementation with *L. reuteri* Inhibitis Melanoma Growth

**Figure 1:**
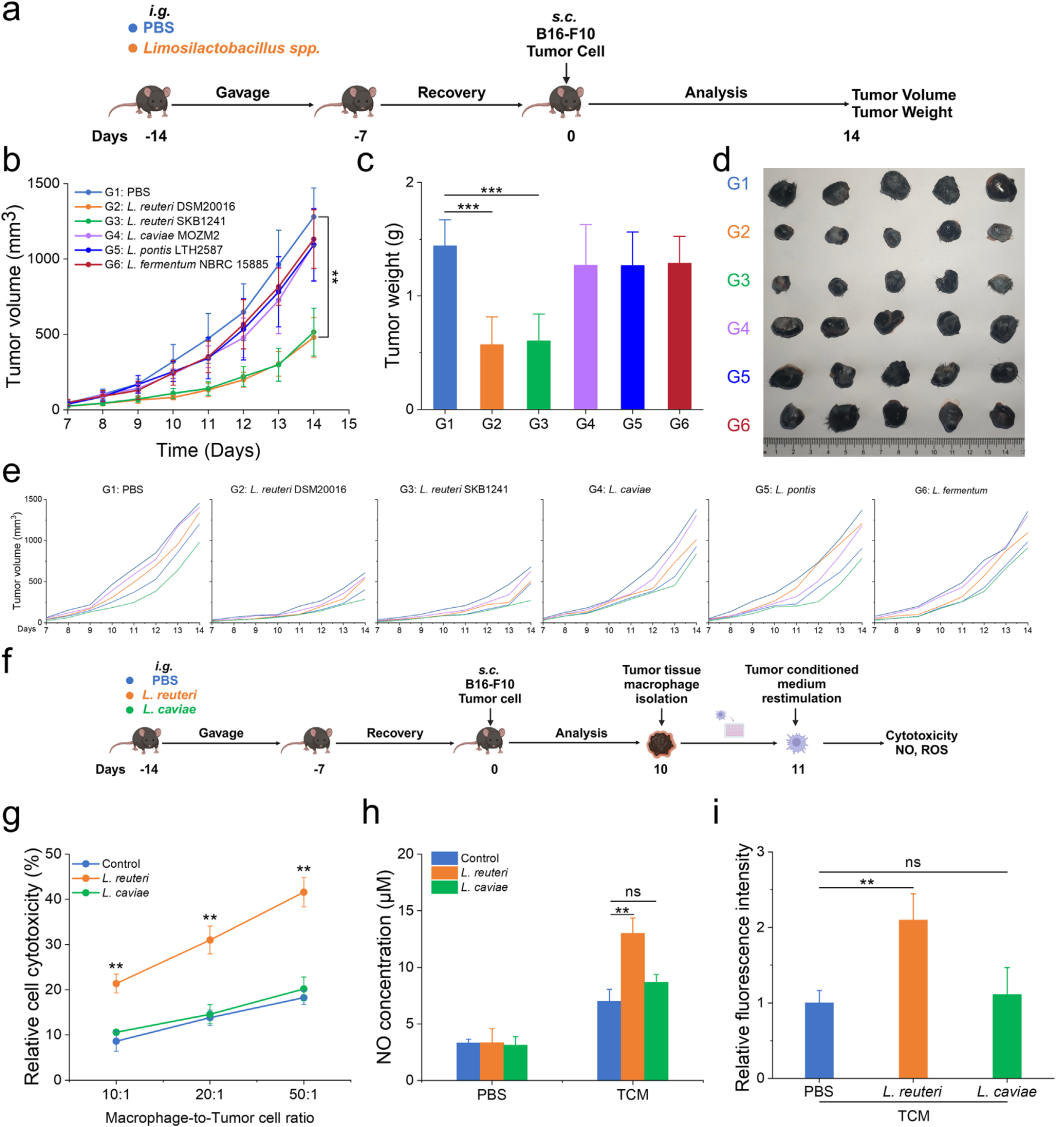
Prophylactic supplementation with *L. reuteri* elicits antitumor immune responses. a) Experimental timeline for evaluating the antitumor effects of *Limosilactobacillus spp*. Mice were gavaged daily with 200 µL of a bacterial suspension containing 2 × 10^8^ CFU of different *Limosilactobacillus* strains for 7 consecutive days. After a 7‐day rest period, B16‐F10 tumor cells were injected into the mice to evaluate the subsequent antitumor responses. b,e) Average tumor volume (b) and individual tumor volumes (e) of melanoma‐bearing mice (*n* = 5). c) Tumor tissue weight (*n* = 5). d) Representative images of tumor tissues (n = 5). f) Experimental design for assessing tumor‐associated macrophage responses after gavage with PBS (control), *L. reuteri*, or *L. caviae*. Mice were gavaged daily with 200 µL of a bacterial suspension containing 2 × 10^8^ CFU of *L. reuteri* or *L. caviae* for 7 consecutive days. After a 7‐day rest period, B16‐F10 tumor cells were injected into the mice, and immune responses of tumor‐associated macrophages were subsequently analyzed. g) Macrophages were cocultured with B16‐F10 cells for 48 h, followed by tumor cell survival assessment via the CCK‐8 assay (*n* = 3). h,i) TNF‐α secretion h) and ROS levels i) in macrophages cultured in medium or B16‐F10 tumor‐conditioned medium (TCM; supernatant collected from B16‐F10 tumor cell culture) (n = 3). Data represent means ± SD from 2–3 independent experiments. ns, not significant; **p < 0.01, ***p < 0.001; NO, nitric oxide; TCM, tumor‐conditioned medium.

The gut microbiota has emerged as a pivotal regulator of host immunity, influencing both cancer progression and immune surveillance.^[^ ^15^ ^]^ Among the array of beneficial microbes, *Limosilactobacillus spp*. have demonstrated considerable promise in modulating host immune responses.^[^ ^16^ ^]^ Here, we assessed whether prophylactic supplementation with *Limosilactobacillus spp*., followed by a washout period, could prime the host immune system before tumor inoculation to enhance antitumor immunity (**Figure** **1**a). Based on phylogenetic analysis of the *Limosilactobacillus* genus, several representative strains were selected for subsequent experimental evaluation to explore their roles in immune activation and antitumor immunity (Figure S1, Supporting Information). Our data demonstrated that preventive supplementation with *Limosilactobacillus reuteri* (*L. re*) DSM20016 and SKB1241 significantly inhibited tumor growth compared to the control group. In contrast, supplementation with *Limosilactobacillus caviae*, *Limosilactobacillus pontis*, or *Limosilactobacillus fermentum* failed to suppress tumor growth (Figure 1b,e). Tumor weight measurements and representative tumor images further corroborated these findings (Figure 1c,d), suggesting that the antitumor effects of *Limosilactobacillus spp*. are species‐specific. We hypothesize that distinct probiotic strains, shaped by divergent phylogenetic lineages, may exert antitumor effects through differential mechanisms.

To elucidate the mechanisms underlying the potent tumor‐suppressive effects of *L. reuteri*, we focused on tumor‐associated macrophages (TAMs), a key immune cell population within the tumor microenvironment (TME) that regulates both tumor progression and immune responses.^[^ ^17^ ^]^ Since macrophage activity is a crucial determinant of antitumor immunity,^[^ ^18^ ^]^ we hypothesized that *L. reuteri* could enhance macrophage‐mediated tumor cytotoxicity. To test this hypothesis, mice were pretreated with PBS, *L. reuteri*, or *L. caviae* as a control (Figure 1f). Our results demonstrated that *L. reuteri* pretreatment significantly increased macrophage cytotoxicity against tumor cells (Figure 1g). Nitric oxide (NO), a critical effector molecule, mediates tumor cell cytotoxicity, enhances macrophage activation, and modulates immune responses.^[^ ^19^ ^]^ Mechanistically, conditioned media from B16‐F10 melanoma cells stimulated macrophages to produce elevated levels of NO and reactive oxygen species (Figure 1h,i). These findings suggest that *L. reuteri* primes macrophages prior to their recruitment into the TME, augmenting their immune response and promoting antimelanoma immunity.

### 2.2 Reuterin Recapitulates the Prophylactic Effect of *L. reuteri* against Melanoma

**Figure 2:**
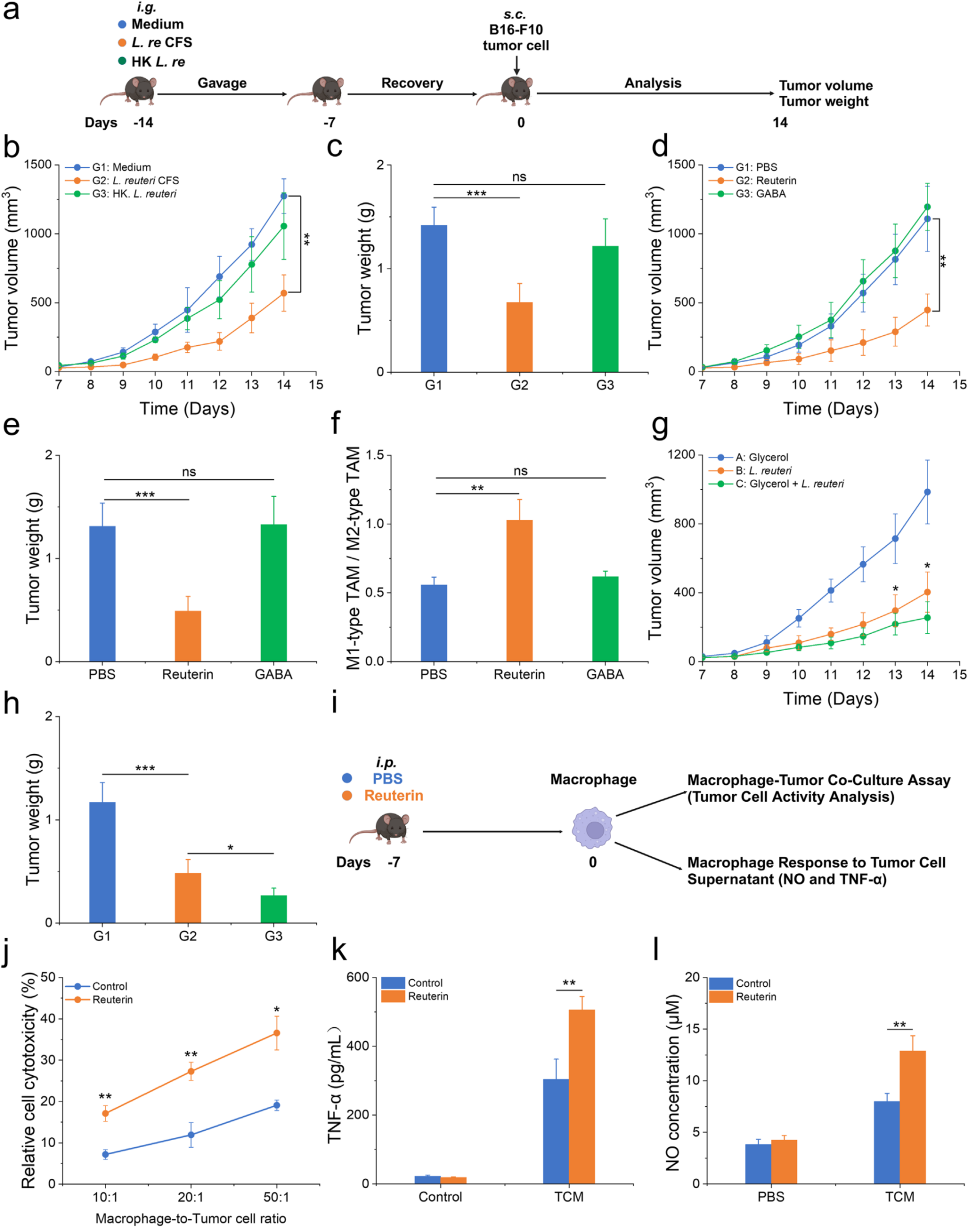
Reuterin recapitulated the prophylactic effect of *L. reuteri*. a) Experimental timeline for evaluating the antitumor effects of *L. reuteri* cell‐free supernatant (CFS) and heat‐killed *L. reuteri* (HK *L. reuteri*). Each group was administered a daily oral gavage of either 200 µL of *L. reuteri* CFS, 200 µL of HK *L. reuteri* suspension (2 × 10^8^ CFU), or 200 µL of MRS medium (control). b) Average tumor volume in melanoma‐bearing mice (*n* = 5). c) Tumor tissue weight with representative images (*n* = 5). d,e) Tumor growth curves d) and tumor weights with representative images e) of melanoma‐bearing mice treated with PBS, GABA, or reuterin (*n* = 5). f) Flow cytometry analysis of the proportions of CD86^+^ (M1 macrophages) and CD206^+^ (M2 macrophages) within CD45^+^F4/80^+^ cells isolated from tumor tissues of B16‐F10 tumor‐bearing mice pretreated with PBS, reuterin, GABA. The M1/M2 macrophage ratio in tumor tissues was analyzed (*n* = 3). g,h) Tumor growth curves g) and tumor weights with representative images h) of melanoma‐bearing mice treated with 5% glycerol, *L. reuteri*, or *L. reuteri* combined with 5% glycerol (*n* = 5). i) Schematic of the experimental setup for evaluating macrophage responses following intraperitoneal injection of PBS (control) or reuterin (10 µg). Macrophages were subsequently isolated from the peritoneal cavity for further analysis. j) Macrophages were cocultured with B16‐F10 cells for 48 h, and tumor cell viability was determined (*n* = 3). k,l) TNF‐α k) and NO l) production by macrophages cultured for 24 h in medium or TCM (*n* = 3). Data represent means ± SD from 2–3 independent experiments. ns, not significant; *p < 0.05, **p < 0.01, ***p < 0.001; NO, Nitric oxide; GABA, γ‐aminobutyric acid; TCM, Tumor‐conditioned medium.

To investigate the underlying mechanisms through which *L. reuteri* enhances macrophage‐mediated antimelanoma immunity, we examined whether its effects are driven by its cellular components or its metabolites. We compared the effects of oral administration of heat‐killed *L. reuteri* and its cell‐free supernatant (CFS), which contains bioactive molecules secreted by the bacteria (**Figure** **2**a). Notably, the administration of *L. reuteri* CFS led to a significant reduction in tumor growth (Figure 2b), as evidenced by decreased tumor weight (Figure 2c). This finding suggests that the tumor‐suppressive effects of *L. reuteri* may be attributed to its secreted metabolites rather than the bacterial cells themselves. Among these metabolites, reuterin—an antimicrobial compound produced by most *L. reuteri* strains—has been identified as a key bioactive molecule.^[^ ^20^ ^]^ Given its immunomodulatory properties, we hypothesized that reuterin plays a pivotal role in mediating the observed antitumor effects. Our analysis showed that among *Limosilactobacillus spp*., only *L. reuteri* strains possess the ability to convert glycerol into reuterin (Figure S2, Supporting Information). This observation raised the question of whether reuterin specifically contributes to the observed tumor‐suppressive effects or if other bioactive compounds might also be involved. To address this, we selected gamma‐aminobutyric acid (GABA)—another metabolite produced by certain *Limosilactobacillus* species—as a control.^[^ ^21^ ^]^ Comparative analysis showed that reuterin significantly inhibited melanoma growth, while GABA had no detectable effect (Figure 2d,e). Further analysis revealed that reuterin supplementation altered the polarization profile of tumor‐associated macrophages. Specifically, flow cytometry revealed a significant increase in CD86^+^ (M1‐like) macrophages and a concurrent decrease in CD206^+^ (M2‐like) macrophages, leading to an elevated M1/M2 ratio (Figure 2f). These results imply that reuterin may, at least in part, mediate its antitumor effects through macrophage‐driven inflammatory remodeling of the tumor microenvironment.

To assess whether glycerol metabolism by *L. reuteri* directly contributes to tumor suppression, we administered *L. reuteri* in a 5% glycerol solution. This combination led to significantly greater tumor inhibition compared to the administration of either glycerol or *L. reuteri* alone (Figure 2g,h). These findings support the hypothesis that reuterin, a key metabolite derived from glycerol,^[^ ^22^ ^]^ is crucial in limiting tumor progression. To further elucidate the role of reuterin in antitumor immunity, we examined its effects on macrophage activity (Figure 2i). Our results showed that reuterin pretreatment significantly enhanced macrophage‐mediated tumor cell killing (Figure 2j), accompanied by increased NO and TNF‐α production upon stimulation with TCM (Figure 2k,l). These findings indicate that reuterin primes macrophages and enhances their response to tumor‐derived signals. Beyond its direct effects on macrophages, we explored whether reuterin modulates antitumor immunity by altering the gut microbiota. Microbial analysis conducted prior to tumor inoculation revealed that reuterin supplementation increased overall diversity, with an enrichment of *Firmicutes* and a reduction in *Bacteroidetes*, as well as an increase in *Lactobacillus* and *Limosilactobacillus* species (Figure S3, Supporting Information). These findings suggest that reuterin enhances immune function by both activating macrophages and modulating the gut microbiota, thereby supporting antitumor immunity.

### 2.3 Reuterin Imprints Innate Memory‐Like Responses in Macrophages to Promote Antimelanoma Activity

**Figure 3:**
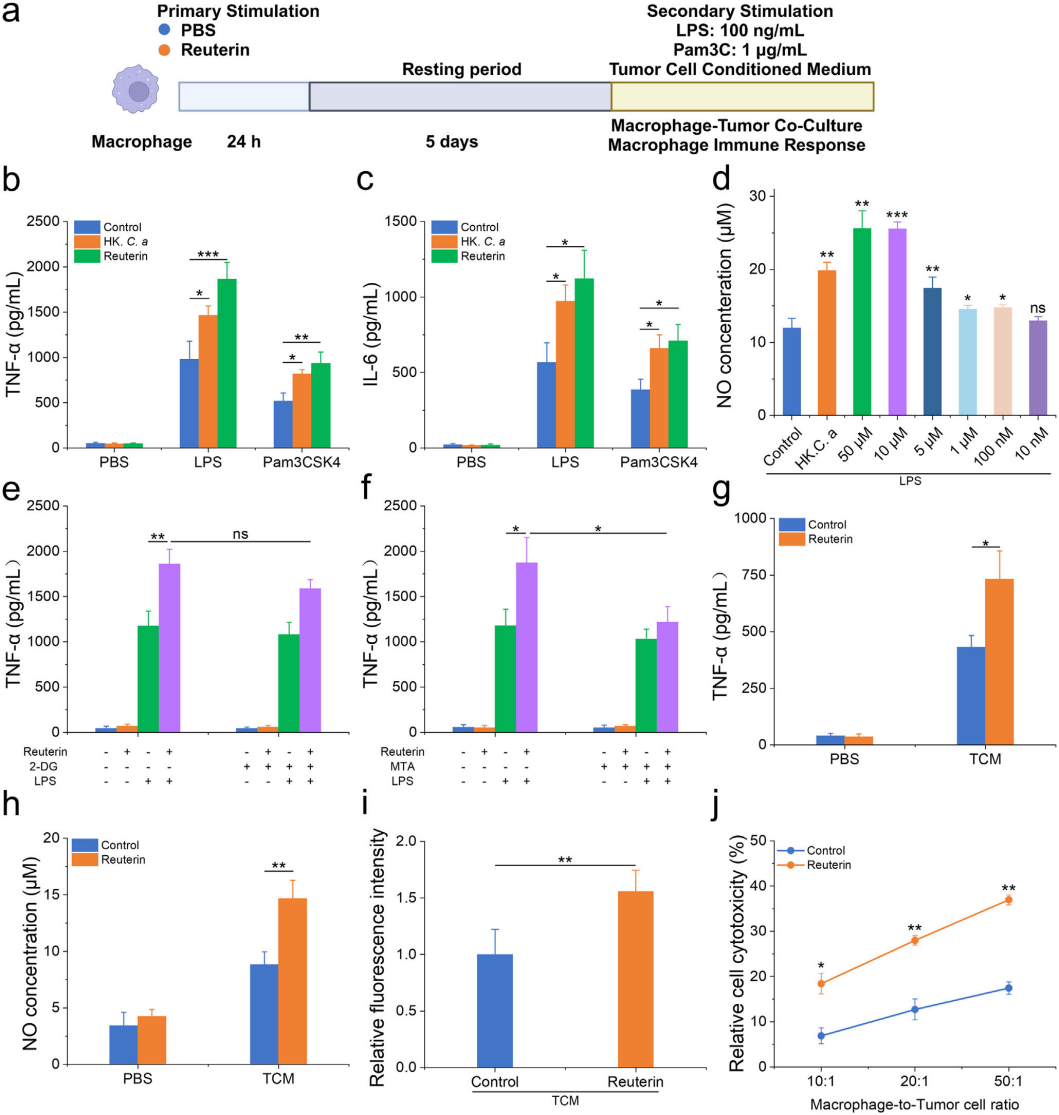
Reuterin triggers trained immunity responses in macrophages to enhance their antimelanoma activity. a) Experimental timeline for assessing macrophage‐trained immunity in vitro. b,c) TNF‐α and IL‐6 secretion in macrophages treated with reuterin (10 × 10^−6^ m) or PBS, followed by 100 ng mL^−1^ LPS or 1 µg mL^−1^ Pam3C stimulation after a 5‐day resting period (*n* = 3). d) NO production in macrophages treated with reuterin (10 nM=10 × 10^−9^ m –50 × 10^−6^ m) or PBS, followed by 100 ng mL^−1^ LPS stimulation after a 5‐day resting period (*n* = 3). e,f) Macrophages were preincubated for 24 h with the glycolysis inhibitor 2‐deoxy‐D‐glucose (2‐DG, 10 × 10^−3^ m) (e) or the histone methyltransferase inhibitor 5′‐deoxy‐5′‐(methylthio)adenosine (MTA, 10 × 10^−6^ m) (f), followed by training with reuterin for 24 h and a 5‐day resting period. TNF‐α levels in the macrophage supernatants were measured after LPS stimulation (*n* = 3). g‐i) TNF‐α g), NO h), and ROS i) production levels in macrophages cultured for 24 h in PBS or TCM (n = 3). j) Macrophages trained with PBS or reuterin were cocultured with B16‐F10 cells for 48 h, and tumor cell viability was determined (*n* = 3). Data represent means ± SD from 3 independent experiments. ns, not significant; *p < 0.05, **p < 0.01, ***p < 0.001; NO, Nitric oxide; TCM, Tumor‐conditioned medium.

Traditionally, the innate and adaptive immune systems are differentiated by their specificity and memory capacity. However, recent studies have shown that the innate immune system is capable of acquiring memory‐like properties after transient stimulation, resulting in an enhanced response to subsequent challenges.^[^ ^23^ ^]^ Research further demonstrates that prevaccination with bacteria‐derived outer membrane vesicles can enhance tumor vaccination through the induction of trained immunity.^[^ ^24^ ^]^ Given the pivotal role of macrophages in tumor progression and immune modulation, we hypothesized that pre‐exposure to reuterin could induce trained immunity in these cells, thereby enhancing their ability to recognize and eliminate tumor cells more effectively, and ultimately strengthening the antitumor immune response. To test this hypothesis, we first evaluated the cytotoxicity of reuterin (Figure S4, Supporting Information), followed by an analysis of its effect on trained immunity activity using our previously established macrophage model (**Figure** **3**a). Macrophages pretreated with reuterin demonstrated enhanced production of TNF‐α and IL‐6 following secondary stimulation with LPS and Pam3CSK4 (Figures 3b,c), similar to the response observed with heat‐killed *C. albicans* (HK *C. a*), a known trained immunity inducer.^[^ ^25^ ^]^ To further explore the functional effects of reuterin on macrophage activation, we evaluated NO levels at various reuterin concentrations following subsequent LPS stimulation (Figure 3d). This effect was also confirmed in bone marrow‐derived macrophages, demonstrating the universality of reuterin‐induced trained immunity in macrophages (Figure S5, Supporting Information). Considering glycolysis as a key metabolic driver of trained immunity, we next examined its role in reuterin‐induced trained immunity.^[^ ^26^ ^]^ However, pretreatment with the glycolysis inhibitor 2‐deoxy‐d‐glucose (2‐DG) failed to impair trained immunity, suggesting that glycolysis is not essential for this process (Figure 3e). Instead, the histone methyltransferase inhibitor 5′‐deoxy‐5′‐(methylthio)adenosine (MTA) significantly attenuated reuterin‐induced trained immunity (Figure 3f), indicating that reuterin drives trained immunity through alternative epigenetic mechanisms independent of glycolysis. Further supporting its functional relevance, macrophages pre‐exposed to reuterin displayed enhanced responsiveness to tumor‐derived signals. Tumor‐conditioned medium stimulation increased TNF‐α, NO, and ROS levels (Figures 3g‐i). In co‐culture experiments, reuterin‐trained macrophages exhibited significantly greater tumor cell cytotoxicity compared to untrained controls (Figures 3j). Collectively, these findings demonstrate that reuterin induces trained immunity, thereby enhancing macrophage‐mediated antitumor responses and strengthening host defense against melanoma.

### 2.4 Reuterin‐Induced Macrophage Trained Immunity via the AHR‐ROS‐HIF‐1α Axis

**Figure 4:**
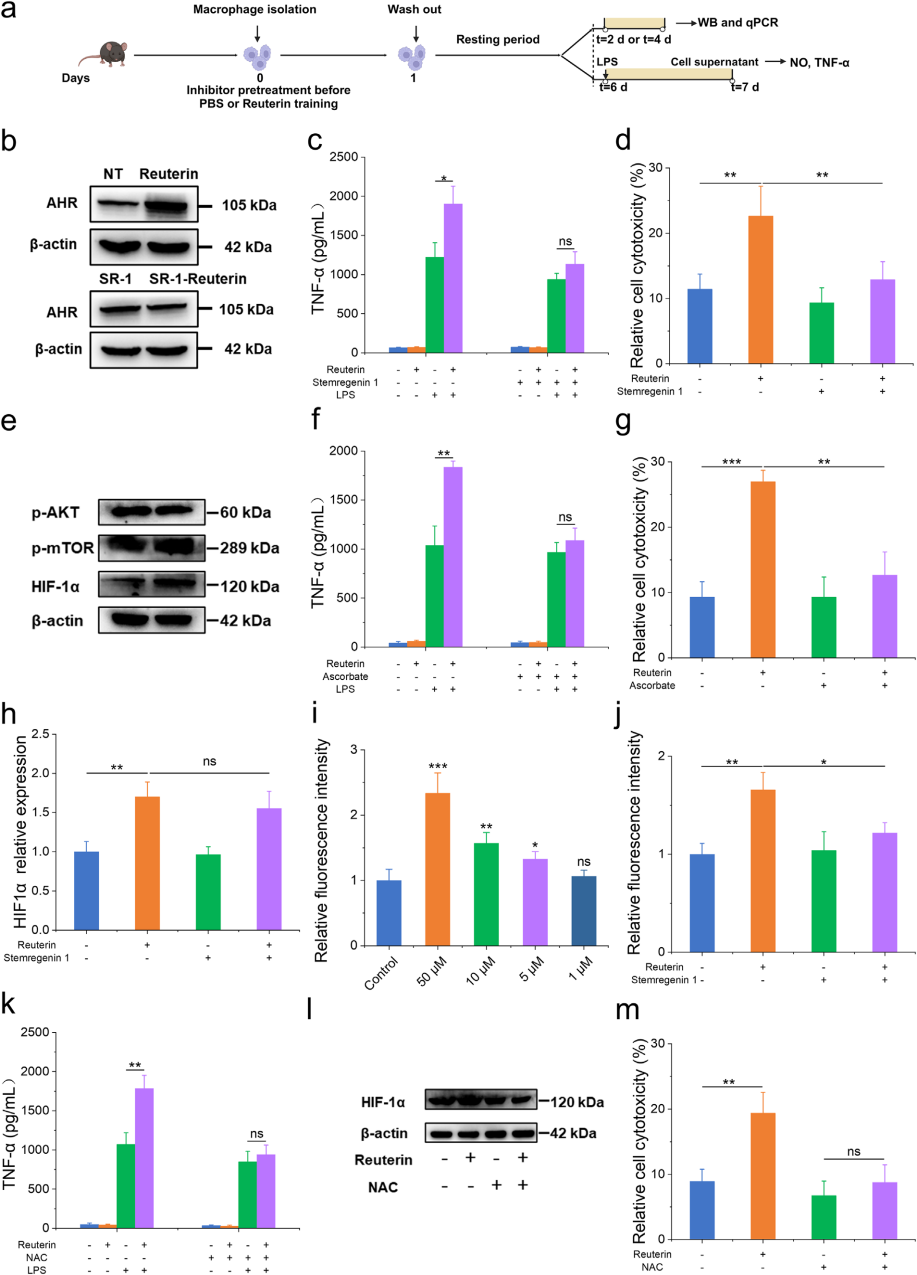
Reuterin enhances macrophage cytotoxicity against tumor cells by inducing trained immunity via the AHR‐ROS‐HIF‐1α axis. a) Experimental timeline for assessing the molecular mechanisms of reuterin‐induced trained immunity in macrophages. b) Western blot analysis of AHR expression in macrophages after reuterin training and a 3‐day resting period. c) TNF‐α levels in macrophages pretreated with the AHR inhibitor stemregenin‐1 (10 × 10^−6^ m), trained with reuterin, rested for 5 days, and stimulated with LPS (100 ng mL^−1^) for 24 h (*n* = 3). d) Tumor cell viability after co‐culture of B16‐F10 cells with macrophages pretreated with stemregenin‐1, trained with reuterin, and rested for 5 days (*n* = 3, 10:1 macrophage‐to‐tumor cell ratio). e) Western blot analysis of p‐AKT, p‐mTOR, and HIF‐1α in macrophages 3 days post‐reuterin training. f) TNF‐α levels in macrophages pretreated with the HIF‐1α inhibitor ascorbate (25 × 10^−6^ m), trained with reuterin, rested for 5 days, and stimulated with LPS (*n* = 3). g) Tumor cell viability after co‐culture of B16‐F10 cells with macrophages pretreated with ascorbate, trained with reuterin, and rested for 5 days (*n* = 3, 10:1 ratio). h) Relative mRNA expression of HIF‐1α in macrophages pretreated with the AHR inhibitor SR‐1 (25 × 10^−6^ m), trained with reuterin, rested for 1 day (*n* = 3). i) ROS levels in macrophages treated with reuterin for 24 h (*n* = 3). j) ROS levels in macrophages pretreated with stemregenin‐1 and trained with reuterin (*n* = 3). k) TNF‐α levels in macrophages pretreated with the ROS inhibitor NAC (1 × 10^−3^ m), trained with reuterin, rested for 5 days, and stimulated with LPS (*n* = 3). l) Western blot analysis of HIF‐1α expression in macrophages pretreated with NAC, trained with reuterin, and rested for 3 days. m) Tumor cell viability after co‐culture of B16‐F10 cells with macrophages pretreated with NAC, trained with reuterin, and rested for 5 days (*n* = 3, 10:1 ratio). Data represent means ± SD from 3 independent experiments. ns, not significant; *p < 0.05, **p < 0.01, ***p < 0.001.

To explore the molecular mechanisms underlying reuterin‐induced trained immunity, we investigated the role of the aryl hydrocarbon receptor (AhR) in macrophages (**Figure** **4**a). Reuterin treatment on day 4 significantly upregulated AhR expression, an effect that was completely reversed by the AhR antagonist stemregenin‐1 (Figure 4b).^[^ ^27^ ^]^ Notably, blocking AhR signaling during the training phase suppressed the enhanced production of TNF‐α and NO upon LPS restimulation (Figures 4c, Figure S6a, Supporting Information). More importantly, AhR inhibition abolished the increased cytotoxicity of trained macrophages against B16‐F10 tumor cells, establishing AhR as a key driver of reuterin‐induced trained immunity (Figure 4d). Given the established role of Akt/mTOR signaling in trained immunity via HIF‐1α induction,^[^ ^28^ ^]^ we examined whether this pathway contributes to reuterin‐induced macrophage training. However, reuterin‐treated macrophages showed no changes in p‐AKT or p‐mTOR levels but exhibited significant upregulation of HIF‐1α protein (Figure 4e). Inhibition of HIF‐1α with ascorbate attenuated the trained immunity phenotype, reducing TNF‐α and NO production (Figure 4f; Figure S6b, Supporting Information) and impairing tumoricidal activity against B16‐F10 cells (Figure 4g). Interestingly, AhR inhibition did not affect HIF‐1α mRNA levels (Figure 4h), suggesting post‐transcriptional regulation of HIF‐1α. To further investigate the molecular basis of HIF‐1α stabilization in trained macrophages, we focused on ROS, which have been implicated in HIF‐1α stabilization, particularly under normoxic conditions.^[^ ^29^ ^]^ Recent studies have shown that ROS produced by reuterin can disrupt *Clostridioides difficile* metabolism and pathogenicity.^[^ ^30^ ^]^ Quantitative analysis revealed that reuterin treatment induced a dose‐dependent increase in ROS levels on day 1 (Figure 4i). Pharmacological inhibition of AhR significantly suppressed ROS production (Figure 4j). Scavenging ROS with N‐acetylcysteine (NAC) notably reduced TNF‐α levels in reuterin‐trained macrophages (Figure 4k), which also decreased HIF‐1α expression (Figure 4l) and impaired the tumoricidal activity of these macrophages (Figure 4m). These results support a causal role for ROS in HIF‐1α activation, emphasizing that the AhR‐ROS‐HIF‐1α axis is crucial for reuterin‐induced trained immunity, driving enhanced macrophage‐mediated antitumor activity.

### 2.5 Glycerophospholipid Metabolism Drives Reuterin‐Induced Trained Immunity

**Figure 5:**
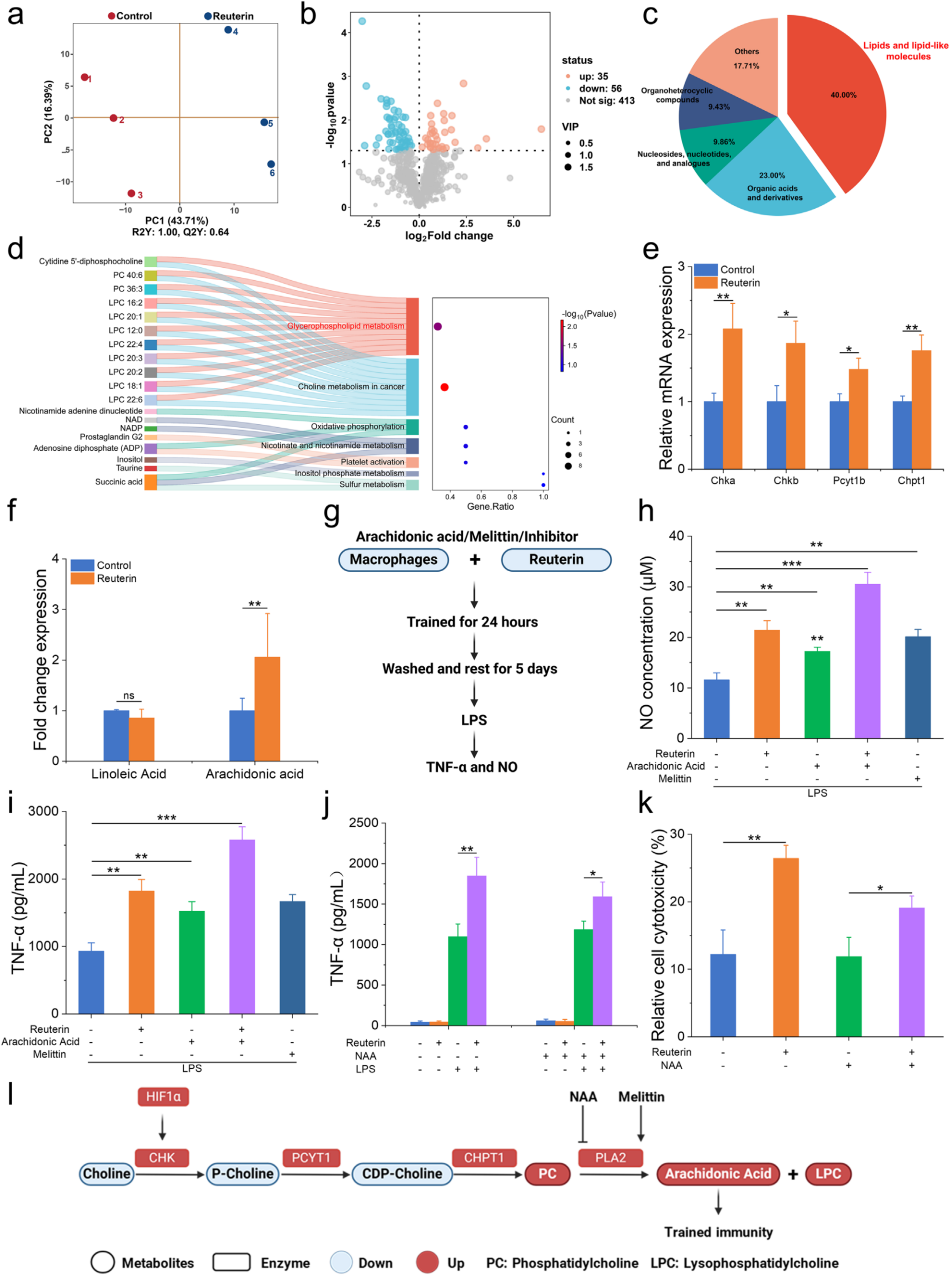
Glycerophospholipid metabolism mediates reuterin‐induced trained immunity. Macrophages were primed with PBS (control) or reuterin for 24 h, followed by a 5‐day resting period. Untargeted metabolomics was performed to assess metabolic changes. a) PLS‐DA plot of metabolomic profiles from PBS‐ and reuterin‐treated macrophages. b) Volcano plot of differential metabolites. c) Classification of differential metabolites. d) KEGG enrichment of differential metabolites (VIP > 1, *P* < 0.05, |log_2_FC| ≥ 1.2). e) Relative mRNA expression levels of glycerophospholipid metabolism genes in PBS‐ or reuterin‐treated macrophages (*n* = 3). f) Linoleic acid and arachidonic acid levels identified in metabolomic profiles. g) Proposed model of trained immunity induced by arachidonic acid and melittin. h, i) NO production (h) and TNF‐α levels i) in reuterin‐trained macrophages supplemented with arachidonic acid (10 × 10^−6^ m) and melittin (250 ng mL^−1^) after LPS restimulation (*n* = 3). j) TNF‐α levels in macrophages pretreated with the PLA2 inhibitor 1‐naphthylacetic acid (NAA; 50 × 10^−6^ m), trained with reuterin, rested for 5 days, and stimulated with LPS (*n* = 3). k) Tumor cell viability (*n* = 3, macrophage:tumor cell ratio = 10:1) following coculture with reuterin‐trained macrophages pretreated with PLA2 inhibitor NAA (50 × 10^−6^ m). l) Schematic diagram of glycerophospholipid metabolism. Data represent means ± SD from 3 independent experiments. ns, not significant; *p < 0.05, **p < 0.01, ***p < 0.001.

The AHR‐ROS pathway regulates macrophage activity and drives HIF‐1α activation, thereby modulating cellular metabolism. To explore the metabolic mechanisms underlying reuterin‐induced trained immunity, we performed nontargeted LC‐MS/MS metabolomics profiling. Principal component analysis (PCA) of 91 differentially regulated metabolites (VIP > 1, *P* < 0.05) revealed distinct metabolic shifts in reuterin‐trained macrophages (**Figure** **5**a,b). Lipids and lipid‐like molecules dominated the metabolic profile, accounting for 40.6% of total metabolites (Figure 5c). KEGG pathway enrichment analysis identified significant enrichment in glycerophospholipid metabolism, choline metabolism, oxidative phosphorylation, and niacin and nicotinamide metabolism, with glycerophospholipid and choline metabolism being the most enriched pathways (Figure 5d). Gene Set Enrichment Analysis (GSEA) further corroborated these findings (Figure S7a,b, Supporting Information). Reuterin treatment reduced key metabolites in choline metabolism, including choline, phosphocholine, and CDP‐choline (Figure S7c–e, Supporting Information). This reduction was attributed to the upregulation of mRNA expression of the metabolic enzymes Chka, Chkb, Pcyt1b, and Chpt1, which mediate choline degradation (Figure 5e). HIF‐1α was found to directly bind the Chka gene promoter,^[^ ^31^ ^]^ enhancing its transcription, further supporting the central role of HIF‐1α in reuterin‐induced trained immunity. Metabolism analysis also revealed that phosphatidylcholine (PC) accumulated due to altered choline metabolism (Figure S7f, Supporting Information), while PC‐derived phosphatidylethanolamine (PE) and phosphatidylserine (PS) levels were reduced (Figure S7g, Supporting Information). Notably, the accumulation of PC provided a substrate for phospholipase A2 (PLA2)‐mediated hydrolysis, leading to increased levels of arachidonic acid and lysophosphatidylcholine (LPC), while linoleic acid remained unchanged (Figure 5f, Figure S7h, Supporting Information). Arachidonic acid metabolites have been shown to modulate gene expression and epigenetic modifications, promoting trained immunity in immune cells.^[^ ^32^ ^]^ Similarly, our previous metabolomics study also demonstrated an increase in arachidonic acid levels in trained immunity induced by heat‐killed *Candida albicans*.^[^ ^25b^ ^]^ To determine whether arachidonic acid contributes to reuterin‐induced trained immunity, we supplemented macrophages with arachidonic acid during reuterin treatment (Figure 5g). As expected, arachidonic acid supplementation enhanced trained immunity, acting synergistically with reuterin to further amplify trained immunity phenotype. Moreover, activation of PLA2 by Melittin^[^ ^33^ ^]^ further enhanced trained immunity (Figures 5h,i), whereas inhibition of PLA2 by 1‐naphthylacetic acid (NAA) partially attenuated reuterin‐induced trained immunity,^[^ ^34^ ^]^ as reflected by a reduction, but not a complete loss, in the tumor cell‐killing capacity, TNF‐α, and NO production (Figure 5j,k, Figure S7i, Supporting Information). These findings highlight that reuterin enhances glycerophospholipid metabolism to promote arachidonic acid biosynthesis, thereby amplifying trained immunity responses (Figure 5k).

### 2.6 Covalent Organic Framework Based Reuterin Induces Trained Immunity in Tumor‐Associated Macrophages

**Figure 6:**
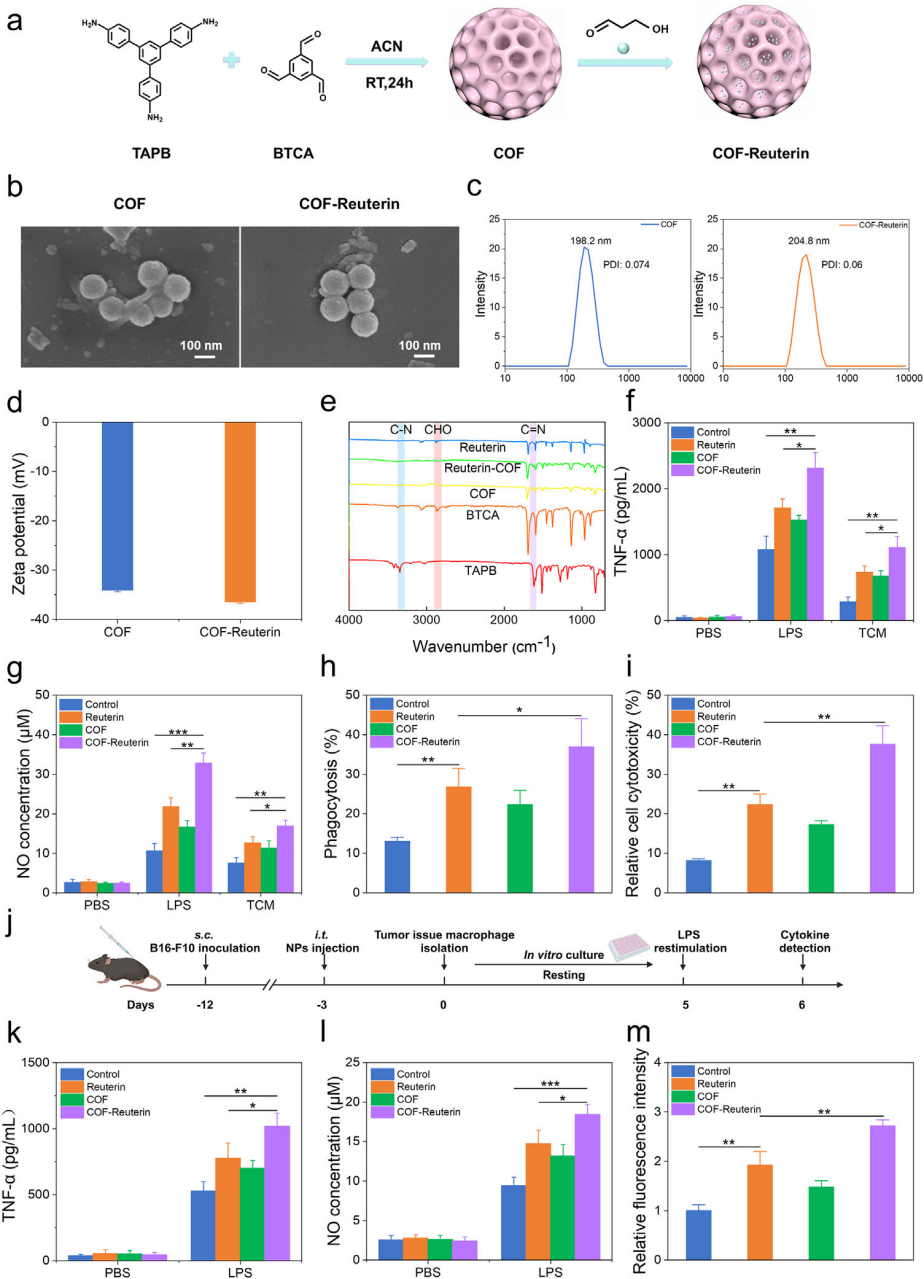
Covalent Organic Framework‐loaded reuterin triggers trained immunity in TAMs. a) Schematic representation of COF‐Reuterin synthesis. b) SEM images of COF and COF‐Reuterin (scale bars: 100 nm). c) Hydrodynamic size distribution of COF and COF‐reuterin (*n* = 3). d) Zeta potential of COF and COF‐Reuterin. e) FTIR spectra confirming COF‐reuterin synthesis. f,g) Macrophages were primed in vitro with COF‐Reuterin or control and stimulated with LPS. NO f) and TNF‐α g) levels were measured (*n* = 3). h) Macrophages were cocultured with *Staphylococcus aureus* for 30 min, and phagocytic activity was assessed by CFU quantification (*n* = 5). i) Macrophages were cocultured with B16‐F10 cells (macrophage: tumor cell ratio 10:1) for 48 h, and tumor cell viability was measured (*n* = 3). j) Schematic diagram illustrating the in vitro trained immunity analysis for TAMs. k,l) TAMs were trained and stimulated with LPS. NO (k) and TNF‐α (l) levels were then measured (n = 3). Data represent means ± SD from 2–3 independent experiments. *p < 0.05, **p < 0.01, ***p < 0.001, ****p < 0.0001. COF, covalent organic framework; TAMs, tumor‐associated macrophages.

To enhance reuterin‐mediated trained immunity and improve antitumor efficacy, we developed a Covalent Organic Framework (COF) delivery system. The COF was synthesized via a Schiff base reaction between 1,3,5‐benzotri‐carbaldehyde (BTCA) and 1,3,5‐tris(4‐aminophenyl)benzene (TAPB) (**Figure** **6**a). Scanning electron microscopy (SEM) revealed monodispersed spherical nanoparticles, with a slight increase in size following reuterin loading (Figure 6b). Dynamic light scattering (DLS) measurements showed hydrodynamic diameters of 198.2 nm (COF) and 204.8 nm (COF‐Reuterin), with polydispersity index (PdI) values of 0.054 and 0.071, respectively (Figure 6c). Zeta potential analysis indicated values of ‐34.1 mV (COF) and ‐36.4 mV (COF‐Reuterin), suggesting good colloidal stability (Figure 6d). Fourier transform infrared spectroscopy (FT‐IR) confirmed successful COF formation and reuterin encapsulation (Figure 6e). In vitro degradation experiments showed that COF‐Reuterin nanoparticles underwent substantial structural breakdown under simulated acidic tumor microenvironment conditions (pH 5.0), with near‐complete disintegration observed at 48 h, as confirmed by scanning electron microscopy (Figure S8, Supporting Information). Biocompatibility assessment revealed low cytotoxicity of COF in macrophages and RAW264.7 cells, compared to Alum (Figure S9, Supporting Information). In addition, hemolysis assays indicated that COF‐Reuterin nanoparticles did not induce red blood cell damage, further supporting their therapeutic safety (Figure S10, Supporting Information). Furthermore, in vivo toxicity assessments conducted post‐administration showed no significant changes in key hepatic and renal biomarkers (ALT, BUN, ALP, CREA, UA), highlighting the nanoparticles’ favorable long‐term safety profile (Figure S11, Supporting Information). To assess whether COF‐Reuterin induces trained immunity, we first examined its impact on macrophage activation. Treatment with COF‐Reuterin notably increased the levels of TNF‐α and NO in response to LPS stimulation (Figure 6f,g). Additionally, COF‐Reuterin‐trained macrophages displayed enhanced phagocytic activity against *Staphylococcus aureus* and improved tumoricidal capacity against B16‐F10 cells (Figure 6h,i). TAMs are the predominant immune cell population within the TME. To determine whether COF‐Reuterin induces trained immunity in TAMs and boosts antitumor activity, we administered various formulations to mice bearing orthotopic B16‐F10 tumors (Figure 6j). TAMs were isolated to assess trained immunity, with COF‐Reuterin inducing significantly higher levels of TNF‐α, NO, and ROS production compared to PBS or free reuterin (Figure 6k‐m). These results demonstrate that COF‐Reuterin effectively induces trained immunity in TAMs, thereby enhancing antitumor responses.

### 2.7 COF‐Reuterin Drives Antigen‐Specific Cellular and Humoral Immune Response

**Figure 7:**
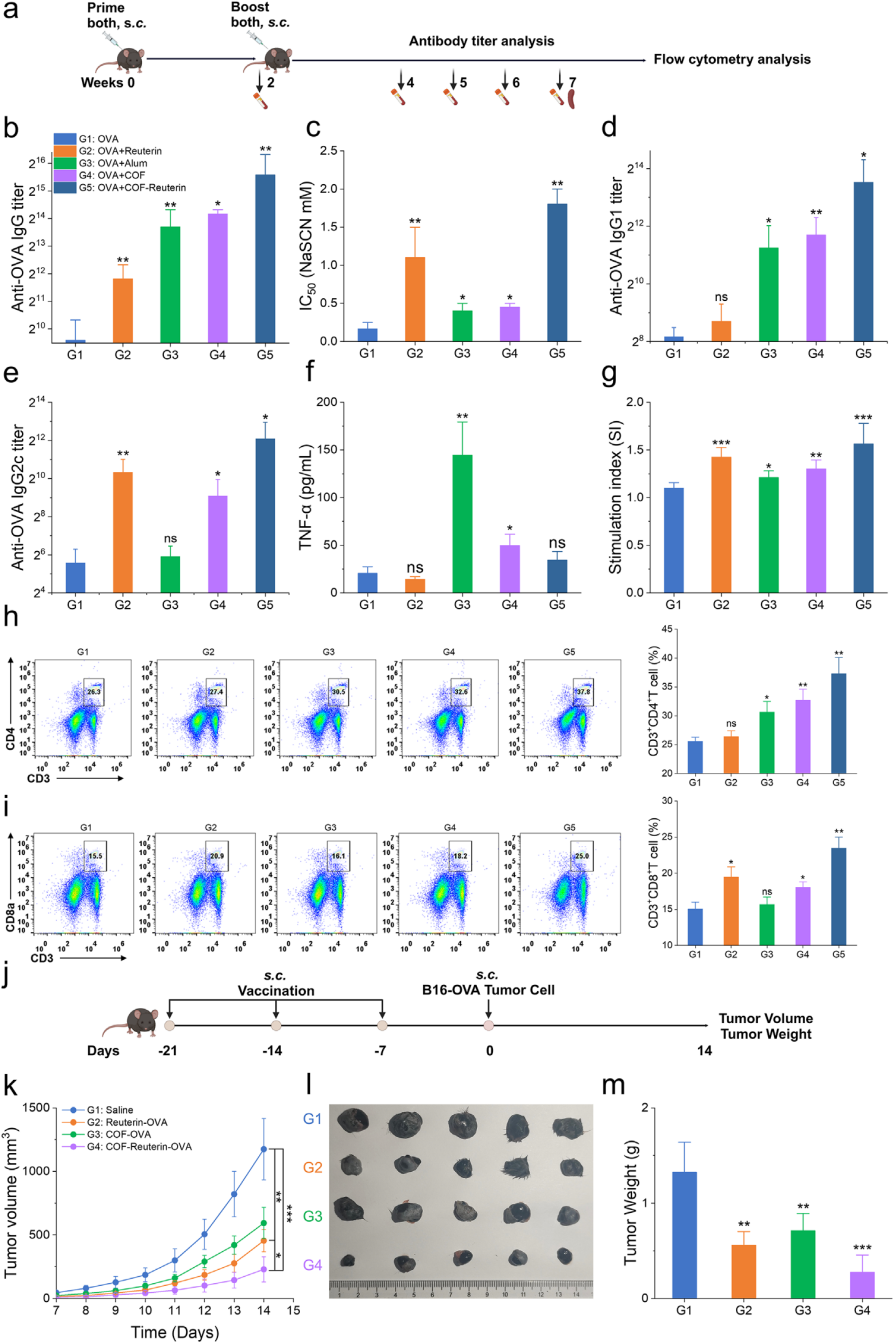
COF‐Reuterin amplifies the antigen‐specific immune response to OVA. a) Schematic representation of the immunization schedule. Mice were subcutaneously immunized with OVA, OVA + Reuterin, OVA + Alum, OVA + COF, OVA + COF‐Reuterin at week 0, followed by a booster dose at week 2. b) Serum levels of anti‐OVA IgG antibodies were quantified by ELISA at week 7 (*n* = 5). c) IgG avidity index was measured at week 4 using NaSCN‐based ELISA (*n* = 5). d, e) Serum levels of anti‐OVA IgG1 d), and IgG2c e) isotype antibodies were quantified by ELISA at week 7 (*n* = 5). f) Serum TNF‐α levels were measured 4 h after vaccination as an early indicator of inflammatory response (*n* = 3). g) At week 7 post‐immunization, splenocytes were stimulated with OVA (100 ng mL^−1^) for 72 h, and cell proliferation was measured (*n* = 3). h, i) Flow cytometry analysis for the detection of CD3^+^ CD4^+^ T cells h) and CD3^+^ CD8^+^ T cells in splenocytes isolated at week 7 from immunized mice. Quantitative analysis results are shown on the right (*n* = 3). j) Schematic schedule for the prophylactic experiments in subcutaneous B16‐OVA tumor‐bearing mice. k) Tumor growth curves of B16‐OVA tumor‐bearing mice (*n* = 5). l) Tumor tissue weight measurement. m) Tumor tissue representative images (*n* = 5). Data represent means ± SD from 2–3 independent experiments. ns, not significant; *p < 0.05, **p < 0.01, ***p < 0.001, ****p < 0.0001.

To evaluate the potential of COF‐Reuterin as a cancer vaccine adjuvant, we employed ovalbumin (OVA) as a tumor‐associated model antigen. Based on its ability to suppress LPS‐induced TNF‐α and NO production in macrophages (Figure S12, Supporting Information), we hypothesized that incorporating reuterin into a vaccine platform could enhance immunogenicity while mitigating excessive inflammatory responses post‐vaccination. To test this hypothesis, we formulated a COF‐Reuterin‐based vaccine and assessed adaptive immune responses in C57BL/6J mice (**Figure** **7**a). COF‐Reuterin + OVA vaccination significantly increased OVA‐specific IgG titers and enhanced the affinity of OVA‐specific antibodies, as measured by ELISA compared to alum‐adjuvanted vaccines (Figure 7b,c). IgG subclass analysis revealed distinct immune polarization: while alum predominantly induced a Th2‐biased response, free reuterin promoted Th1 polarization. COF‐Reuterin effectively balanced Th1/Th2 responses, elevating both IgG1 and IgG2c, with a more pronounced increase in IgG2c compared to alum (Figure 7d,e). Post‐vaccination cytokine analysis showed no significant elevation in systemic TNF‐α levels (Figure 7f). Safety assessments revealed no organ abnormalities or significant weight changes, supporting the biocompatibility of COF‐Reuterin (Figure S13, Supporting Information). Flow cytometry analysis demonstrated that COF‐Reuterin +OVA vaccination increased CD4⁺ and CD8⁺ T cell proportions compared to OVA alone or OVA + alum (Figure 7h,i). Further, OVA re‐stimulation revealed robust antigen‐specific lymphocyte proliferation in the COF‐Reuterin group (Figure 7g), indicating that COF‐Reuterin effectively activates an immune response specific to the OVA antigen. To assess its protective efficacy, mice were immunized weekly with saline, Reuterin‐OVA, COF‐OVA, or COF‐Reuterin‐OVA for three consecutive weeks (Figure 7j), followed by a B16‐OVA tumor challenge. COF‐Reuterin‐OVA exhibited superior antitumor efficacy, significantly reducing tumor burden, as evidenced by decreased tumor volume and weight (Figure 7k–m, Figure S14, Supporting Information). Notably, COF‐loaded reuterin treatment significantly increased the proportion of CD86^+^ (M1‐like) macrophages while decreasing CD206^+^ (M2‐like) macrophages within the tumor microenvironment, indicating a shift toward a proinflammatory macrophage phenotype (Figure S15a–c, Supporting Information). To evaluate the post‐training immune response induced by COF‐Reuterin, macrophages were isolated from tumor tissues of immunized mice from all groups and restimulated with LPS. Macrophages from the COF‐Reuterin group showed enhanced production of the proinflammatory cytokine TNF‐α upon restimulation, consistent with features of trained immunity (Figure S15d, Supporting Information). These findings establish COF‐Reuterin‐OVA as an effective prophylactic vaccine candidate, eliciting potent antitumor immunity.

### 2.8 COF‐Reuterin Exhibits Potent Anticancer Activity and Remodels the Tumor Microenvironment

**Figure 8:**
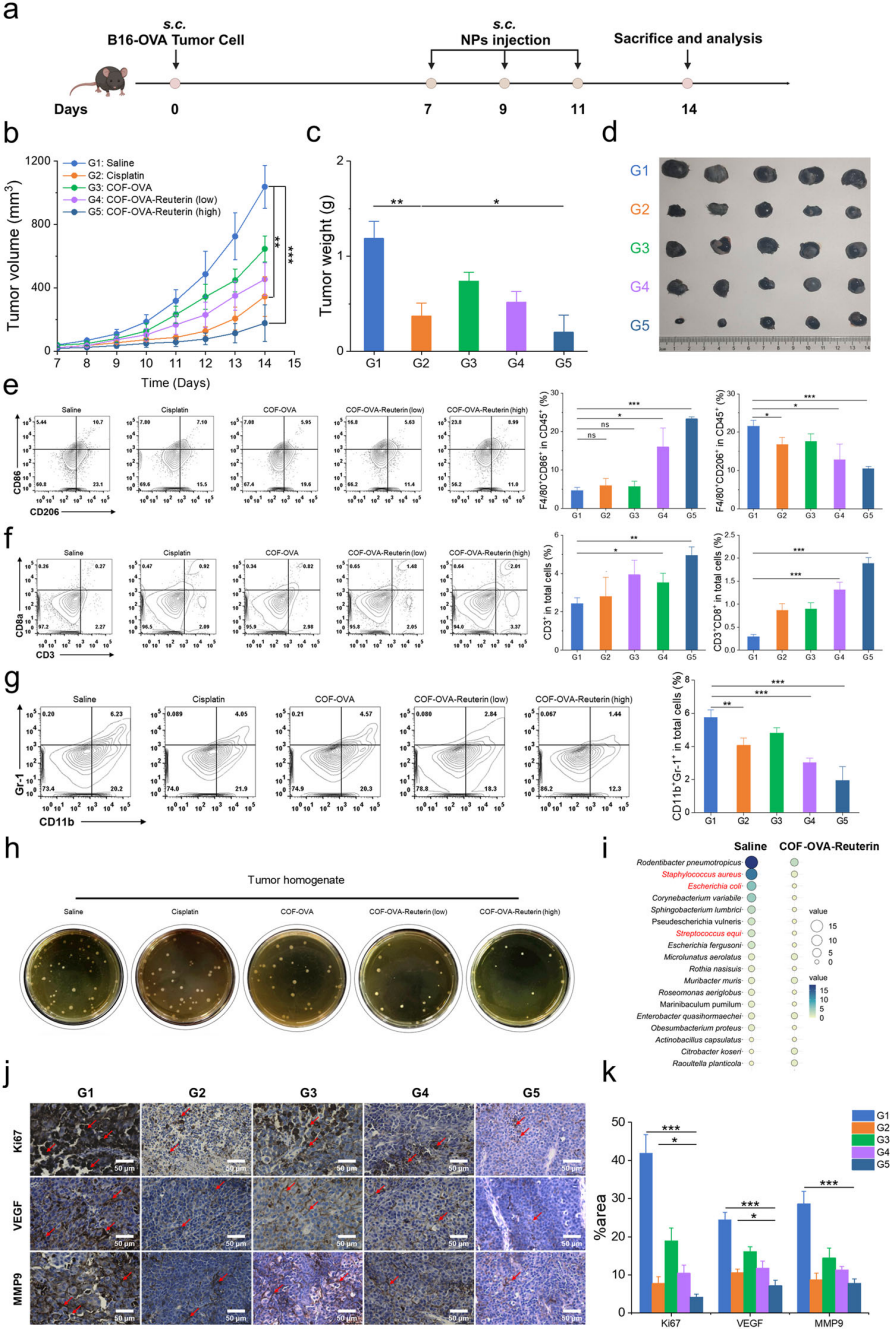
COF‐Reuterin enhances antitumor efficacy and remodels the tumor microenvironment. a) Schematic representation of the experimental timeline for anticancer studies in subcutaneous B16‐OVA tumor‐bearing mice. b) Tumor growth curves of B16‐OVA tumor‐bearing mice treated with different formulations (*n* = 5). c) Tumor tissue weight quantification (*n* = 5). d) Tumor tissue representative images (n = 5). e) Proportion of F4/80^+^CD86^+^ in CD45^+^ cells (M1 macrophages) and F4/80^+^CD206^+^ in CD45^+^ cells (M2 macrophages) in the tumor tissues of B16‐OVA tumor‐bearing mice (*n* = 3). f) Proportion of CD3^+^ and CD3^+^CD8^+^ among total tumor‐infiltrating cells (*n* = 3). g) Proportion of CD11b^+^ and Gr‐1^+^ myeloid‐derived suppressor cells (MDSCs) among total tumor‐infiltrating cells (*n* = 3). h) Representative images of bacterial cultures from tumor tissues grown on tryptic soy broth (TSB) plates. i) Bubble plot showing the relative abundance of bacterial species in tumor tissues of B16‐OVA tumor‐bearing mice treated with saline or COF‐OVA‐Reuterin. Bubble size represents bacterial abundance, with key species highlighted in red significantly reduced in the COF‐Reuterin‐OVA group. j) Immunohistochemical analysis of melanoma tissues demonstrated the expression of Ki67, VEGF, and MMP9 following treatment (scale bar, 50 µm). k) Quantitative analysis of Ki67, VEGF‐A, and MMP9 expression was performed by measuring the percentage of positive staining in tumor tissues (*n* = 3). Data represent means ± SD from 2–3 independent experiments. ns, not significant; *p < 0.05, **p < 0.01, ***p < 0.001.

The therapeutic efficacy of COF‐Reuterin was assessed in B16‐OVA tumor‐bearing mice (**Figure** **8**a). Consistently, COF‐Reuterin‐OVA exhibited the most potent antitumor activity, achieving 85.93% tumor inhibition compared to the PBS group (Figure 8b–d and Figure S16, Supporting Information), highlighting its potential as a therapeutic cancer vaccine. Notably, COF‐Reuterin‐OVA exhibited a superior therapeutic effect compared to cisplatin, as reuterin directly induced tumor cell cytotoxicity more efficiently, leading to a more significant reduction in tumor growth (Figure S17, Supporting Information). No significant body weight loss was observed during treatment (Figure S18, Supporting Information), further supporting the excellent biocompatibility of COF‐Reuterin‐OVA. To further investigate its immunomodulatory effects, we analyzed tumor‐infiltrating immune cells post‐vaccination. Flow cytometry revealed a significant increase in F4/80⁺CD86⁺ M1 macrophages, along with a concurrent reduction in F4/80⁺CD206⁺ M2 macrophages (Figure 8e). This shift toward an elevated M1/M2 macrophage ratio is indicative of a more pro‐inflammatory, antitumor microenvironment (Figure S19, Supporting Information). Consistently, COF‐Reuterin‐OVA‐treated tumors displayed enhanced T‐cell infiltration, with a marked increase in CD3⁺ and CD8⁺ cytotoxic T cells compared to cisplatin (Figure 8f). Moreover, COF‐Reuterin significantly reduced CD11b⁺Gr‐1⁺ myeloid‐derived suppressor cells (MDSCs), alleviating immunosuppression and improving immune surveillance (Figure 8g). Biosafety is a fundamental consideration in vaccine development, requiring thorough evaluation prior to in vivo application. Histopathological analysis using H&E staining revealed no pathological abnormalities in major organs, further supporting their sustained biocompatibility (Figure S20, Supporting Information).

Recognizing that intratumor dysbiosis contributes to immune tolerance and immunotherapy resistance,^[^ ^35^ ^]^ we next assessed COF‐Reuterin's impact on the tumor microbiota. Colony‐forming unit (CFU) assays revealed a fivefold reduction in total bacterial load following treatment, with 16S rRNA sequencing confirming significant depletion of *Rodentibacter pneumotropicus*, *Staphylococcus aureus*, and *Escherichia coli* within the TME (Figure 8h,i). Notably, in vitro assays demonstrated that COF‐Reuterin‐OVA effectively inhibited the growth of *Staphylococcus aureus*, and *Escherichia coli*, suggesting a direct antimicrobial effect, which may contribute to intratumor bacterial remodulating (Figure S21, Supporting Information).^[^ ^36^ ^]^ Immunohistochemical analysis revealed that COF‐Reuterin‐OVA treatment significantly reduced the expression of Ki67 (a proliferation marker), VEGF‐A (vascular endothelial growth factor A), and MMP9 (matrix metalloproteinase 9) in melanoma tissues, indicating its effective inhibition of tumor cell proliferation, angiogenesis, and invasiveness (Figure 8j,k). To further validate the generalizability of our findings beyond melanoma, we evaluated tumor growth kinetics and tumor weight in an orthotopic 4T1 breast tumor model (Figure S22a, Supporting Information). Notably, intravenous administration of antibiotics alone was insufficient to inhibit tumor growth, whereas the combination of cisplatin and antibiotics demonstrated a significantly enhanced antitumor effect (Figure S22b‐d, Supporting Information). Consistent with our melanoma findings, treatment with COF‐Reuterin‐TAs significantly inhibited tumor progression in this breast cancer model. These findings indicate that COF‐Reuterin exerts its therapeutic effects through multiple mechanisms, including macrophage polarization, T‐cell activation, suppression of immunosuppressive cells, tumor microbiota modulation, and direct tumor cell cytotoxicity. Collectively, these immunomodulatory and antimicrobial properties contribute to enhancing antitumor immunity and improving therapeutic outcomes.

## 3 Discussion

Over the past two decades, significant breakthroughs have been achieved in the field of cancer immunotherapy. Immune checkpoint blockade (ICB) therapy and chimeric antigen receptor T (CAR‐T) cell therapy, as advanced immunotherapeutic approaches, have demonstrated remarkable therapeutic efficacy across a variety of tumor types. However, 30–70% of patients fail to respond to immune checkpoint inhibitors, and the performance of CAR‐T cell therapy against malignant solid tumors has been less than optimal.^[^ ^37^ ^]^ Therefore, there is an urgent need for innovative strategies that can convert immunologically “cold” tumors into “hot” tumors with immune reactivity. Recent research has introduced the concept of “immune mobilization strategies,” which aim to induce trained immunity, thereby enhancing the efficiency of the innate immune system over time.^[^ ^24^ ^]^ This elevated state enables the immune system to mount a stronger response upon subsequent immune stimulation. Inducing trained immunity in TAMs to activate and amplify antitumor immune responses represents an innovative strategy with the potential to overcome the immunosuppressive TME, offering new breakthroughs in cancer immunotherapy.

The relationship between cancer and the gut microbiota has garnered increasing attention in recent years, revealing complex interactions that can influence tumorigenesis and treatment outcomes.^[^ ^38^ ^]^ However, our understanding of how microbial metabolites affect the initiation and progression of melanoma remains limited. Recent studies have highlighted the dual role of certain bacterial populations in either promoting or inhibiting cancer growth.^[^ ^39^ ^]^ Microbial byproducts, such as butyrate and other short‐chain fatty acids, have been shown to exert diverse effects on cancer cells. While some studies suggest that high concentrations of butyrate can induce cell death in cancer cells,^[^ ^40^ ^]^ other research indicates that carbohydrate‐derived metabolites, like butyrate, can promote excessive proliferation of colonic epithelial cells, increasing the risk of colorectal tumors.^[^ ^41^ ^]^ In the present study, we identified a unique antimelanoma effect of *Limosilactobacillus reuteri*, a species within the *Limosilactobacillus* genus, and demonstrated that its metabolite reuterin plays a critical role in inducing trained immunity, which is essential for the antimelanoma response.

Trained immunity is characterized by the epigenetic and metabolic reprogramming of innate immune cells in response to specific stimuli. This rewiring not only confers broad, nonspecific protection against a variety of diseases but also enhances the immune response to established immunotherapies and vaccines. While much of the current research on trained immunity has focused on biologically derived molecules like β‐glucan and BCG vaccines, there is growing evidence that other molecular inducers, such as uremic toxin indoxyl sulfate, also play a role in modulating immune responses. Specifically, indoxyl sulfate has been shown to induce trained immunity via the AhR‐dependent arachidonic acid pathway.^[^ ^42^ ^]^ Interestingly, while many other molecules, such as hippuric acid, indole 3‐acetic acid, and kynurenic acid, are AhR ligands, they did not show trained immunity responses like indoxyl sulfate.^[^ ^42^ ^]^ This suggests that specific molecular characteristics may determine the induction of trained immunity. Additionally, indoxyl sulfate promotes ROS production through the aryl hydrocarbon receptor‐NADPH oxidase pathway.^[^ ^43^ ^]^ Previous studies have identified the Akt/mTOR/HIF‐1α signaling pathway as a key mediator in the glycolytic induction of trained immunity. However, our results demonstrate that in reuterin‐induced trained immunity, the phosphorylation levels of AKT and mTOR remain largely unchanged. In contrast, we observed a notable increase in HIF‐1α expression. Interestingly, we also observed that reuterin‐induced training in macrophages results in a substantial accumulation of ROS. ROS have been shown to act as alternative inducers of HIF‐1α, independent of hypoxia, promoting HIF‐1α stabilization and facilitating the metabolic reprogramming essential for trained immunity. Furthermore, studies have demonstrated that ROS generated during trauma‐induced injury and tumor progression play a crucial role in stabilizing HIF‐1α responses,^[^ ^44^ ^]^ highlighting their importance in immune reprogramming.

Glycolysis, glutamine metabolism, and cholesterol synthesis pathways are known to be essential for β‐glucan‐induced trained immunity.^[^ ^45^ ^]^ However, no significant alterations were observed in these pathways in our metabolomics data. In contrast, glycerophospholipid metabolism and choline metabolism in cancer cells were significantly activated, leading to increased choline consumption and phosphatidylcholine accumulation, which correlated with elevated HIF‐1α levels. Phosphatidylcholine is hydrolyzed by phospholipase A2 to produce both arachidonic acid and lysophosphatidylcholine.^[^ ^46^ ^]^ Previous studies have shown that the glycerophospholipid‐linoleic acid metabolic pathway plays a key role in BCG vaccine‐induced trained immunity.^[^ ^47^ ^]^ Our findings indicate that phosphatidylcholine accumulation plays a critical role in the induction of trained immunity. Moreover, we demonstrated that arachidonic acid, the hydrolysis product of phosphatidylcholine, contributes to the induction of trained immunity. Recent studies have pinpointed various endogenous sterile damage factors, such as oxidized low‐density lipoprotein (oxLDL), lipoprotein(a), uric acid, hyperglycemia, and Western dietary patterns, which induce trained immunity through epigenetic reprogramming in human monocytes.^[^ ^48^ ^]^ Our results further support the idea that enhancing reuterin‐induced trained immunity through the activation of arachidonic acid synthesis or direct supplementation with arachidonic acid may represent an effective strategy. This suggests that the signaling pathway could serve as a potential target for trained immunity‐based immunotherapies, with arachidonic acid or its metabolic agonists potentially serving as vaccine adjuvants.

Trained immunity, a functional state of myeloid cells, has emerged as a promising target in immune oncology. Research has demonstrated that inducing trained immunity with a bone marrow‐avid nanoimmunotherapeutic effectively inhibits tumor growth and enhances the immune system's responsiveness to checkpoint blockade therapy.^[^ ^49^ ^]^ Furthermore, studies indicate that nanovaccine formulation induces trained innate immunity and elicits strong antitumor adaptive immunity, significantly reversing tumor growth and even completely eliminating established tumors.^[^ ^50^ ^]^ Probiotics are increasingly recognized for their potential in cancer immunotherapy, with demonstrated immunomodulatory effects across various cancers. Trimethylamine N‐oxide (TMAO) derived from *Clostridiales* induces pyroptosis in tumor cells, promoting cell death and enhancing CD8^+^ T cell‐mediated antitumor immunity.^[^ ^51^ ^]^ Engineered *Escherichia coli* Nissle 1917‐derived L‐Arginine increases tumor‐infiltrating T cells in colorectal cancer, boosting immune activation and enhancing the efficacy of PD‐L1 blocking antibodies.^[^ ^52^ ^]^ Additionally, butyric acid from *Ruminococcus* and related genera activates cytotoxic CD8^+^ T cells in gastric cancer, improving the response to anti‐PD1 therapy.^[^ ^53^ ^]^ These findings suggest that probiotics, by modulating immune function and the tumor microenvironment, may offer novel strategies for cancer immunotherapy as adjuvant therapies.

Porous materials, such as metal‐organic frameworks (MOFs) and Covalent Organic Frameworks (COFs), have demonstrated efficacy in drug delivery by encapsulating drugs and enabling sustained release. Specifically, Zeolitic Imidazolate Framework‐8 (ZIF‐8) nanoparticles, functionalized with polydopamine (PDA), have been incorporated into a dual‐network hydrogel composite, which is designed to accelerate the healing of diabetic bone defects.^[^ ^54^ ^]^ COFs are crystalline organic polymers connected by dynamic covalent bonds, which allow them to load small molecules. Their metal‐free composition is expected to enhance biocompatibility.^[^ ^55^ ^]^ Hydrogels are biocompatible materials with flexible properties that make them useful for both sensing and therapy. They can be designed to assemble in response to disease, degrade in a controlled way, and release drugs locally in response to stimuli.^[^ ^56^ ^]^ A biphasic system combining hydrogels and nanoparticles can simultaneously deliver antigens and immune training inducers, enhancing both adaptive and trained immunity for long‐lasting antitumor immune memory in cancer vaccines.^[^ ^50^ ^]^ Hydrogels can modulate the balance between M1 and M2 macrophage phenotypes, thereby suppressing inflammation induced by reactive oxygen species.^[^ ^54^, ^57^ ^]^ Macrophages trained with COF‐Reuterin exhibit significantly increased tumoricidal activity, demonstrating enhanced efficacy in killing tumor cells. Inducing trained immunity in innate immune cells holds great promise for cancer therapy by amplifying the antitumor immune response. However, whether trained immunity can be activated in TAMs within the TME to trigger antitumor immune responses remains underexplored. Macrophages exhibit metabolic plasticity, allowing them to reprogram their metabolic pathways in response to environmental cues. The bioactive and smart‐responsive composite hydrogel can selectively induce macrophage polarization toward the M2 phenotype by modulating the local microenvironment to promote tissue repair and regeneration.^[^ ^58^ ^]^ M1‐TAMs are effective at phagocytosis and cytokine secretion, whereas M2‐TAMs promote immune suppression and tumor evasion. Our results show that COF‐Reuterin increases the proportion of M1‐TAMs and decreases M2‐TAMs in melanoma, along with a significant reduction in myeloid‐derived suppressor cells (MDSCs). These findings underscore the potential of COF‐Reuterin‐induced trained immunity as a novel strategy to reprogram TAMs, offering a promising approach to enhance antitumor immunity and improve cancer therapy.

Despite the relatively low abundance of resident microbes within tumors, they play an essential role in tumor progression.^[^ ^59^ ^]^ These microbes are primarily localized within tumor and immune cells, where they modulate cellular functions and activities. Each tumor has a unique bacterial community composition. For example, *Staphylococcus aureus* is a common pathogen in breast tumors and melanoma.^[^ ^35b^ ^]^ Although the precise contributions and molecular mechanisms of tumor‐associated microbes in tumorigenesis remain poorly understood, existing evidence indicates that antibiotic treatment can effectively reduce bacterial presence in tumor tissues, thereby inhibiting the progression of lung cancer.^[^ ^60^ ^]^ Additionally, bacterial depletion in breast tumors has been shown to significantly decrease metastasis, highlighting the potential of targeting tumor‐associated microbes as a therapeutic strategy.^[^ ^61^ ^]^ In line with this, our results demonstrate that COF‐Reuterin exerts a multifaceted effect within the tumor microenvironment. Firstly, it enhances antitumor immunity by inducing trained immunity and improving the immunosuppressive tumor microenvironment. Secondly, COF‐Reuterin directly targets the tumor microbiota through the bactericidal activity of reuterin, resulting in a significant reduction in bacterial load within the tumor. Lastly, by leveraging the cytotoxic properties of reuterin, COF‐Reuterin holds promise for reversing established tumor growth and may even contribute to the complete eradication of tumors.

In summary, we identified a gut commensal *Limosilactobacillus reuteri*‐derived small molecule trained immunity agonist, reuterin, which enhances glycerophospholipid metabolism through the AHR‐ROS‐HIF‐1α signaling axis. This leads to increased arachidonic acid production, thereby inducing trained immunity and promoting the host's ability to suppress melanoma growth in mice. In vivo experiments confirmed the tumor therapeutic efficacy of COF‐Reuterin, demonstrating its capacity to induce trained immunity, reduce intratumor bacterial load, and enhance tumor cell killing. Consequently, our study validates the successful strategy of inducing trained immunity in TAMs for melanoma treatment in mice. These findings provide valuable strategies for the development of tumor vaccines that do not rely on specific antigens, offering the potential for broad‐spectrum vaccine efficacy and guiding the development of next‐generation cancer vaccines.

## 4 Experimental Section

### Bacteria Strains and Oral Administration

The *Limosilactobacillus* genus strains *L. reuteri* DSM 20 016, *L. reuteri* SKB1241, *L. caviae* MOZM2, *L. pontis* LTH 2587, and *L. fermentum* NBRC 15 885 were maintained at −80 °C in 25% glycerol prior to use. For experimental preparation, the strains were subcultured twice in MRS broth (Hopebio, China) and incubated overnight at 37 °C. Following cultivation, bacterial cells were harvested and resuspended in PBS to a final concentration of 1 × 10^9^ CFU mL^−1^. Mice were then gavaged daily with 200 µL of the bacterial suspension of different *Limosilactobacillus* strains for 7 consecutive days as indicated in the experimental protocol.

### Mice

Female C57BL/6J mice (6–8 weeks old) were purchased from Liaoning Changsheng Biotechnology Co., Ltd. and maintained in specific pathogen‐free (SPF) conditions at the Laboratory Animal Center of Jilin University. Mice were randomly assigned to experimental groups, ensuring age and sex matching.

### Trained Immunity Experimental Protocol

For in vitro experiments, Peritoneal macrophages (PMs) were isolated from 8‐week‐old female C57BL/6J mice by lavage of the peritoneal cavity. The cells were exposed to 10 × 10^−6^ m reuterin for 24 h, with heat‐killed *Candida albicans* (HK‐*C. a*.) as a positive control. After incubation, the cells were washed twice with PBS and cultured in RPMI 1640 medium supplemented with 10% fetal bovine serum (FBS) for 5 days. On day 6, the cells were restimulated with PBS, LPS (100 ng mL^−1^), Pam3C (1 µg mL^−1^), or 50% conditioned medium from B16‐F10 tumor cells. For in vivo experiments, 8‐week‐old female C57BL/6J mice were orally administered PBS, live *L. reuteri*, or heat‐killed *L. reuteri* (2 × 10⁸ CFU) daily for 7 days. For inhibitor experiments, cells were pretreated with stemregenin 1 (MCE, 10 × 10^−6^ m), or Ascorbic acid (MACKLIN, Shanghai, China, 25 × 10^−6^ m) for 24 h prior to exposure to reuterin. For nanoparticle experiments, 8‐week‐old C57BL/6J mice were injected intraperitoneally with PBS, COF, or COF‐Reuterin. Seven days post‐injection, PMs were isolated and restimulated as described above.

### Prophylactic and Therapeutic Antitumor Effects

In the prophylactic study, 7‐week‐old female C57BL/6 mice were subcutaneously vaccinated with saline, OVA (100 µg), Reuterin (100 µg), COF‐OVA (100 µg COF + 100 µg OVA), or COF‐OVA‐Reuterin (100 µg COF‐Reuterin + 100 µg OVA) per mouse. Vaccination was administered once a week for three weeks, followed by a challenge with 5 × 10^5^ B16‐OVA cells. Tumor progression was monitored daily using caliper measurements, and tumor volume was calculated using the formula V = (length × width2) / 2. In the therapeutic study, 7‐week‐old female C57BL/6 mice were injected with B16‐OVA cells. When the tumor volume reached ≈50 mm^3^, mice were treated with saline (control), cisplatin (80 µg), COF‐OVA (100 µg COF + 100 µg OVA), COF‐OVA‐Reuterin (Low) (50 µg COF‐Reuterin + 100 µg OVA), or COF‐OVA‐Reuterin (High) (250 µg COF‐Reuterin + 100 µg OVA). Treatments were administered on days 7, 9, and 11 post‐inoculation. Mice were euthanized on day 14 for tumor analysis. In the breast tumor therapy study, 8‐week‐old female BALB/c mice were orthotopically injected with 4T1 cells to establish the tumor model. When tumor volumes reached ≈30–50 mm^3^, mice were randomly assigned to receive one of the following treatments: saline (control); intravenous administration of 300 µL antibiotic suspension containing vancomycin (10 mg mL^−1^), imipenem/cilastatin (4 mg mL^−1^), and neomycin (1.5 mg mL^−1^); intravenous cisplatin (80 µg); COF combined with tumor antigens derived from 4T1 tumor cell membranes (COF‐TAs; 250 µg COF + 100 µg tumor cell membranes); or COF‐TAs combined with Reuterin (COF‐Reuterin‐TAs; 250 µg COF‐Reuterin + 100 µg tumor cell membranes). Treatments were administered on days 1, 4, 7, and 14 post‐randomization. Tumor volumes were measured every three days throughout the duration of the study.

### 16S rRNA Gene Sequencing

Fecal genomic DNA was extracted using a commercial DNA extraction kit, followed by library construction and high‐throughput sequencing. The 16S rRNA gene library was constructed by amplifying the V3‐V4 regions, with forward primer 5′‐ACTCCTACGGGAGGCAGCA‐3′ and reverse primer 5′‐CGGACTACHVGGGTWTCTAAT‐3′. Data analysis was performed using QIIME2 and R (v3.3.2), with amplicon sequence variants (ASVs) classified using the Silva 138 reference database. Alpha diversity, including Shannon's index and observed species, was assessed via the Wilcoxon test and visualized with ggplot2. Beta diversity was evaluated using Bray‐Curtis dissimilarity and visualized through principal coordinate analysis (PCoA). Taxonomic composition at the phylum and family levels was visualized using QIIME2's taxa barplot function.

### Metabolome Analysis

Macrophages were treated with PBS or reuterin (10 × 10^−6^ m) for 24 h and rested for 5 days. Metabolites were extracted using a methanol‐acetonitrile‐water solution (2:2:1, v/v) and analyzed by UHPLC‐MS on a Vanquish system coupled to an Orbitrap Exploris 120. Chromatography was conducted on a Waters ACQUITY UPLC BEH Amide column with mobile phases A (25 mmol L^−1^ ammonium acetate and ammonia hydroxide, pH 9.75) and B (acetonitrile). The Orbitrap operated in IDA mode with full MS and MS/MS resolutions of 60 000 and 15 000, respectively. Data processing was performed using Compound Discoverer 3.3, and statistical analysis was conducted with R and Python.

### Macrophage‐Mediated Cytotoxicity Assay

The cytotoxic activity of macrophages against B16‐F10 melanoma cells was assessed using a Transwell co‐culture system (Corning, USA). B16‐F10 cells were seeded into the lower chamber of a Transwell plate at a density of 2 × 10⁴ cells per well in DMEM containing 10% fetal bovine serum (FBS) and incubated overnight to allow adherence. Activated macrophages were added to the upper chamber (0.4 µm pore inserts) in RPMI 1640 medium supplemented with 10% FBS. The co‐culture was maintained for 48 h under standard conditions. After the incubation period, the lower chamber was washed twice with PBS to remove debris and nonadherent cells. Tumor cell viability was determined using the Cell Counting Kit‐8 (CCK‐8, Beyotime, China) according to the manufacturer's protocol.

### Nitric Oxide Production

Nitric oxide (NO) levels in cell culture supernatants were quantified using a NO assay kit (Beyotime) in accordance with the manufacturer's instructions. Supernatants were collected post‐treatment, and NO production was evaluated by the Griess reaction, which measures nitrite, a stable end product of NO metabolism. Absorbance was recorded at 540 nm using a microplate reader, and the data were normalized to a standard curve derived from known sodium nitrite concentrations.

### ROS Measurement

Macrophages were seeded in 96‐well plates at a density of 2 × 10⁵ cells per well and treated with reuterin or inhibitor for 24 h. After treatment, the medium was removed, and the cells were washed with PBS. The cells were then incubated with 10 × 10^−6^ m DCFH‐DA in RPMI 1640 at 37 °C for 30 minutes. Following incubation, the cells were washed with PBS and ROS levels were assessed by fluorescence detection, using excitation and emission wavelengths of 488 nm and 525 nm, respectively.

### RT‐qPCR Analysis

RNA was extracted from cells using TRIzol reagent (TaKaRa) according to the manufacturer's instructions, and complementary DNA (cDNA) was synthesized using the PrimeScript Reverse Transcriptase Kit (TaKaRa). Quantitative real‐time PCR (RT‐qPCR) was performed with SYBR Green reagents on a QuantStudio 3 Detection System (Applied Biosystems, Thermo Fisher Scientific). Gene expression levels were normalized to β‐actin as the internal control and calculated using the comparative cycle threshold (2^−ΔΔCt^) method. Primer sequences are listed in Table S1 (Supporting Information). Each sample was analyzed in technical triplicates to ensure data reliability.

### Reuterin Detection

Reuterin production was quantified as described by Circle et al.^[^ ^62^ ^]^ Briefly, overnight cultures of *Limosilactobacillus* strains were harvested by centrifugation to collect the cells. The cells were washed twice with 50 × 10^−6^ m potassium phosphate buffer (PBS) and resuspended in 25 mL of 250 × 10^−6^ m glycerol, corresponding to 500 mg of the cells. The suspension was incubated at 37 °C for 2 h to allow reuterin production. After incubation, the cells were pelleted by centrifugation, and the supernatant was filtered through a 0.45 µm syringe filter. For reuterin detection, 300 µL of each supernatant was mixed with 225 µL of a 10 × 10^−6^ m tryptophan solution, followed by the addition of 900 µl of concentrated HCl. The reaction mixtures were incubated at 37 °C for 20 minutes, and absorbance was measured at 560 nm using a Biotek microplate reader.

### Synthesis of COF‐Reuterin

The COF was synthesized using a Schiff base condensation reaction. Briefly, 1 mg of 1,3,5‐tris(4‐aminophenyl)benzene (TAPB) and 2 mg of benzene‐1,3,5‐tricarbaldehyde (BTCA) were dissolved in 4 mL of acetonitrile with ultrasonic assistance. Glacial acetic acid (0.3 mL) was added, and the mixture was stirred at room temperature for 24 h. After 12 h, 100 µL of reuterin solution was added, and the reaction continued for another 12 h. The product was isolated by centrifugation at 10 000 rpm for 10 min, washed three times with anhydrous ethanol, and dried at 60 °C for 24 h, yielding a pale yellow powder (COF‐Reuterin). Reuterin was dissolved in DMSO to prepare a stock solution, serially diluted for HPLC analysis, and used to generate a standard curve of peak area versus concentration to determine drug loading and encapsulation efficiency in the nanoparticles. To evaluate acidic degradation, 1 mg of COF‐Reuterin was dispersed in 4 mL of PBS (pH 5.0) and incubated at 37 °C with shaking (220 rpm). Samples (0.2 mL) were taken at 0, 4, 8, 24, and 48 h for morphological observation by scanning electron microscopy.

### Trained Immunity Assay for TAMs

Tumor‐associated macrophages (TAMs) were isolated following a previously established protocol^[^ ^63^ ^]^ Briefly, B16‐F10 tumor‐bearing mice were administered various nanoparticle formulations, after which tumor tissues were harvested. The tumors were finely minced and enzymatically digested with collagenase type IV at 37 °C for 1 h. A single‐cell suspension was obtained by passing the digested tissue through a 70‐µm cell strainer. The cells were incubated with red blood cell lysis buffer at 4 °C for 2 minutes, followed by washing with (PBS). The resuspended cells were mixed with 40% Percoll solution and carefully layered with 80% Percoll solution. After centrifugation at 750 × *g* for 30 min, the macrophage‐enriched population at the interphase was collected and cultured in RPMI‐1640 medium supplemented with 10% fetal bovine serum (FBS) and 1% penicillin‐streptomycin. Following an overnight incubation, fresh medium was replenished for subsequent stimulation and trained immunity analysis.

### Statistical Analysis

Data are presented as means ± standard deviation (SD). All statistical analyses and graphical representations were conducted using GraphPad Prism 9.0 software. The Shapiro–Wilk test was employed to evaluate the normality of the data distribution.The F‐test was used to evaluate whether the variances between the two groups were equal, while the paired t‐test with unequal variances was applied to compare the means of the two groups. The Brown–Forsythe test was used to assess the homogeneity of variances across multiple groups. Depending on the data distribution, independent‐samples Student's *t*‐test, one‐way ANOVA or two‐way ANOVA was used to compare two or more groups. Significance was determined at *p < 0.05, **p < 0.01, ***p < 0.001.

## Conflict of Interest

The authors declare no conflict of interest.

## Ethics declarations

All in vivo experiments were performed in accordance with the institutional guidelines and regulations for animal experimentation of Jilin University. The protocols were approved by the Jilin University Animal Center's Ethics Committee (Approval No. KT201902111).

## Supporting information



Supporting Information

## Data Availability

The data that support the findings of this study are available from the corresponding author upon reasonable request.
