# Supplementary material for: Covalent Organic Frameworks‐Delivered Reuterin Drives Trained Immunity in Tumor‐Associated Macrophages to Enhance Melanoma Immunotherapy via Glycerophospholipid Metabolism
Source: Adv Sci (Weinh). 2025 Jun 30;12(36):e04784. doi: 10.1002/advs.202504784 (PMC12462988; doi:10.1002/advs.202504784)
Supplement: Supplementary file 1 — Supporting Information [file ADVS-12-e04784-s001.docx]

# Supporting Information

**Covalent Organic Frameworks-Delivered Reuterin Drives Trained Immunity in Tumor-Associated Macrophages to Enhance Melanoma Immunotherapy via Glycerophospholipid Metabolism**

*Jian-Gang Zhang*†*, Xiao-Mei Zhang*†*, Xi Wu*†*, Cheng-Kai Zhou, Zhen-Zhen Liu, Xue-Yue Luo, Liang-Zhang, Wei Chen*, Yong-Jun Yang**

ZJG, ZXM, WX, ZCK, LZZ, LXY, ZL, CW, YYJ

College of Veterinary Medicine, Jilin University, Changchun Jilin Province, 130062 P. R. China

†These authors contributed equally to this work.

*Corresponding authors: Yong-Jun Yang (youngjune@jlu.edu.cn); Wei Chen, (chw_cc@jlu.edu.cn)

**
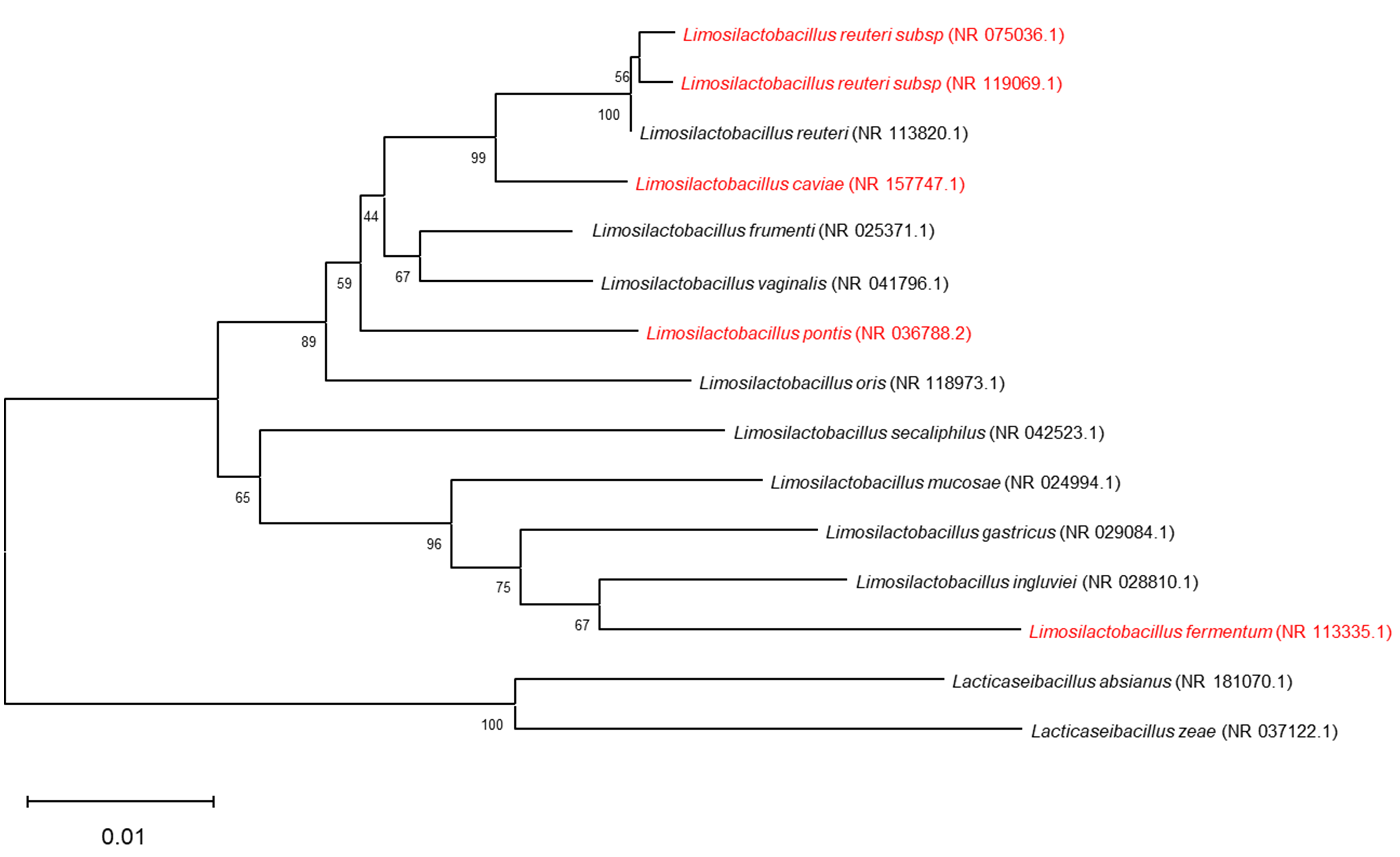
**

**Figure S1.** A phylogenetic tree inferred from 16S rRNA gene sequences using the NJ method depicts the relationship between *Limosilactobacillus reuteri* and other species within the *Limosilactobacillus* genu

**
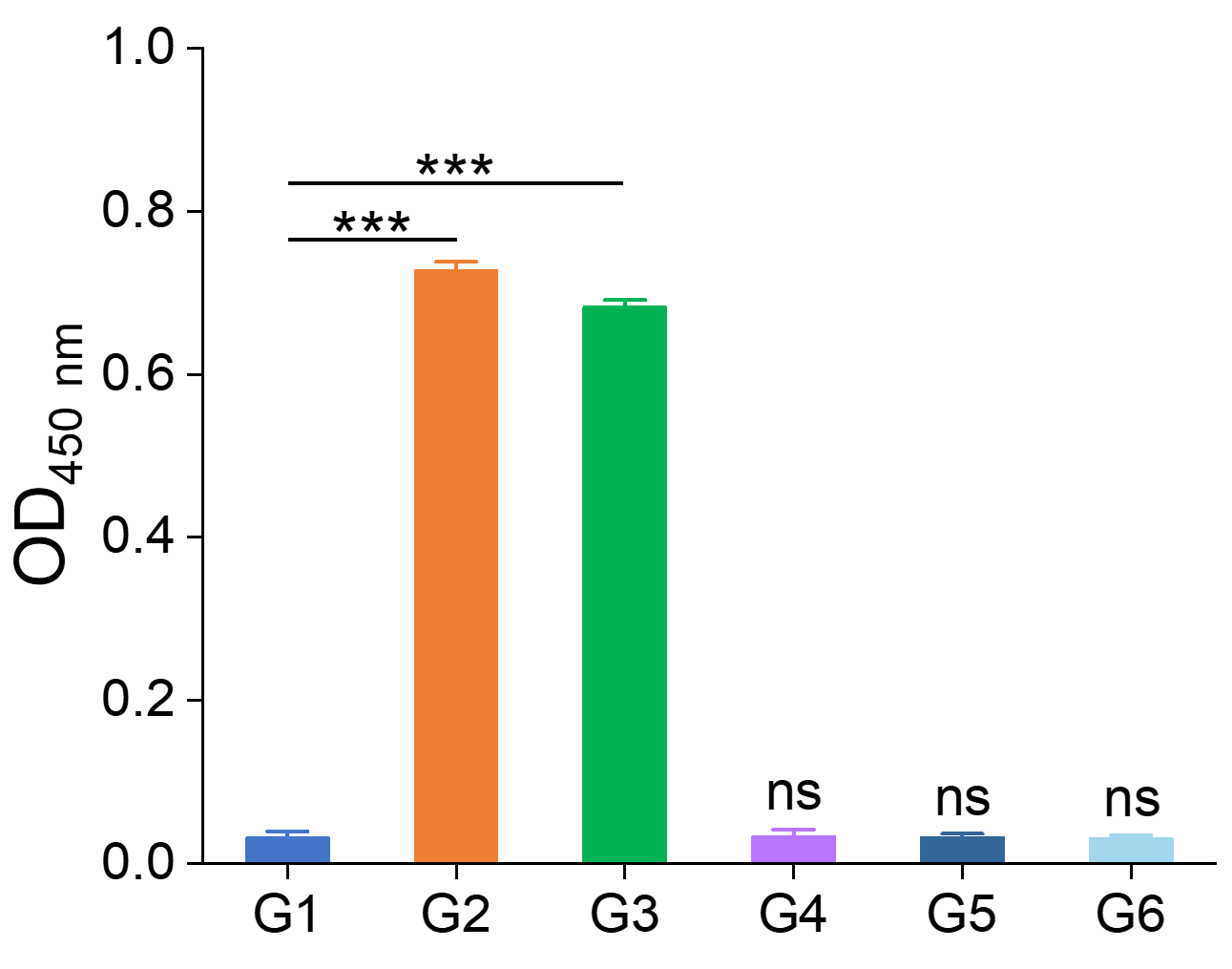
**

**Figure S2.** Evaluation of *Limosilactobacillus spp.* strains for their ability to convert glycerol into reuterin (n = 3). Data are presented as means ± SD. ****p* < 0.001.

**
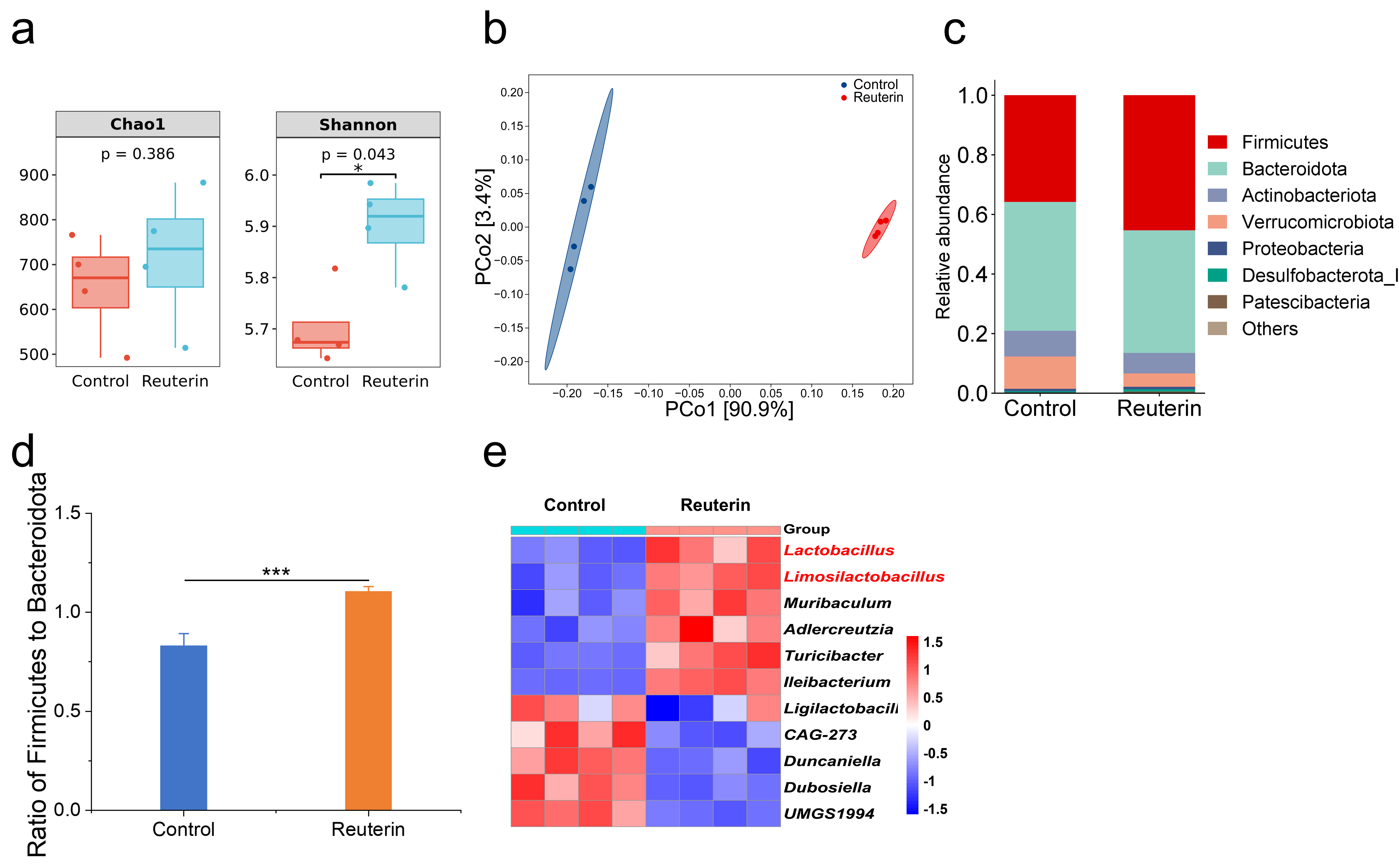
**

**Figure S3.** Modulation of gut microbiota composition following reuterin supplementation. a) Chao1 and Shannon index of gut microbiota in mice treated with PBS or reuterin. b) Principal coordinates analysis (PCoA) of gut microbial communities based on Bray-Curtis dissimilarity. c) Relative abundance of gut microbiota at the phylum level. d) Ratio of Firmicutes to Bacteroidota in the gut microbiota. e) Heatmap showing the differential abundance of key bacterial genera in the gut microbiota (n = 4). Highlighted genera include *Lactobacillus*, *Limosilactobacillus*. Data are presented as means ± SD. ****p* < 0.001.

**
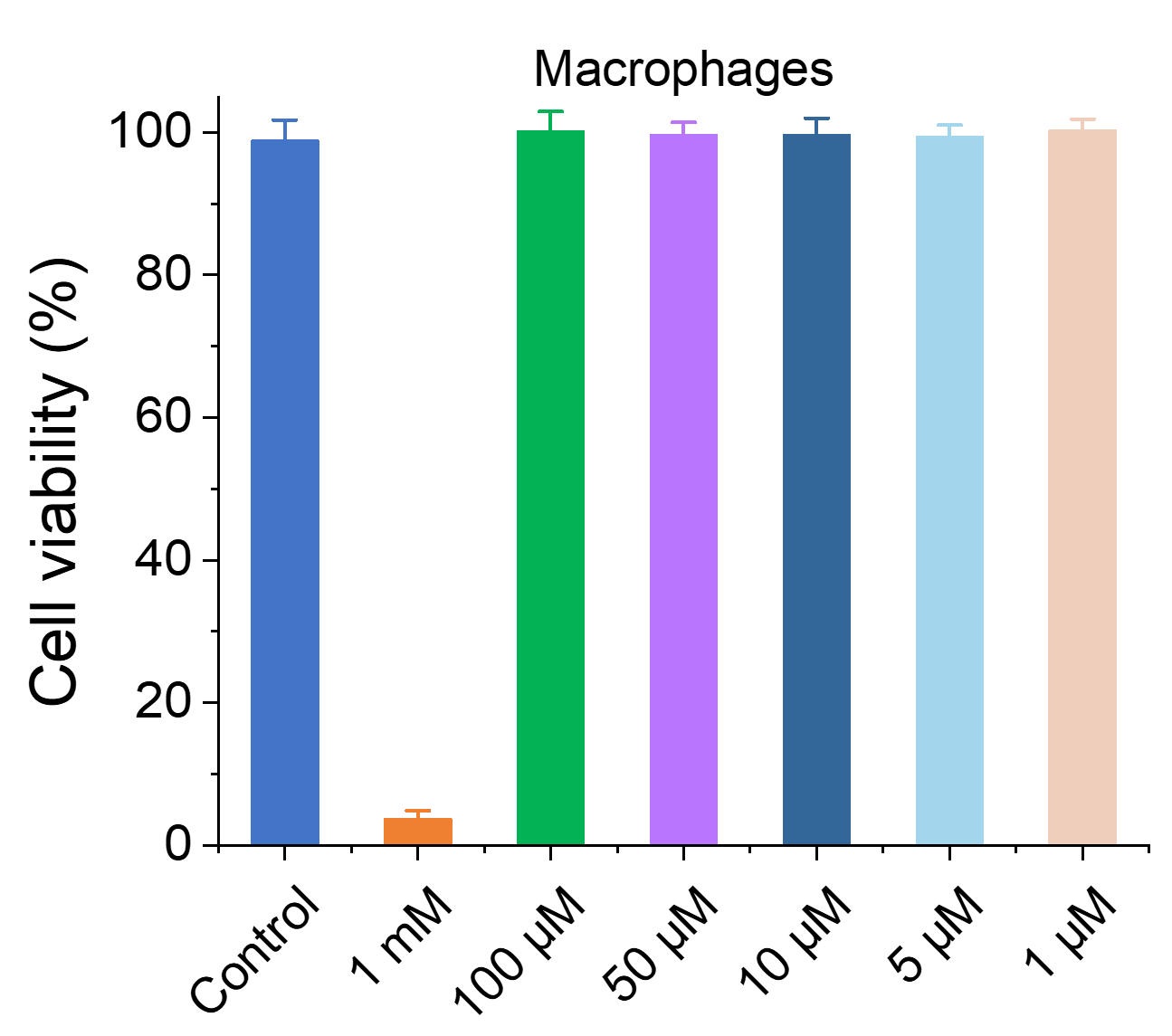
**

**Figure S4.** Cytotoxicity analysis of reuterin on macrophages (n = 3). Data represent means ± SD from 3 independent experiments.

**
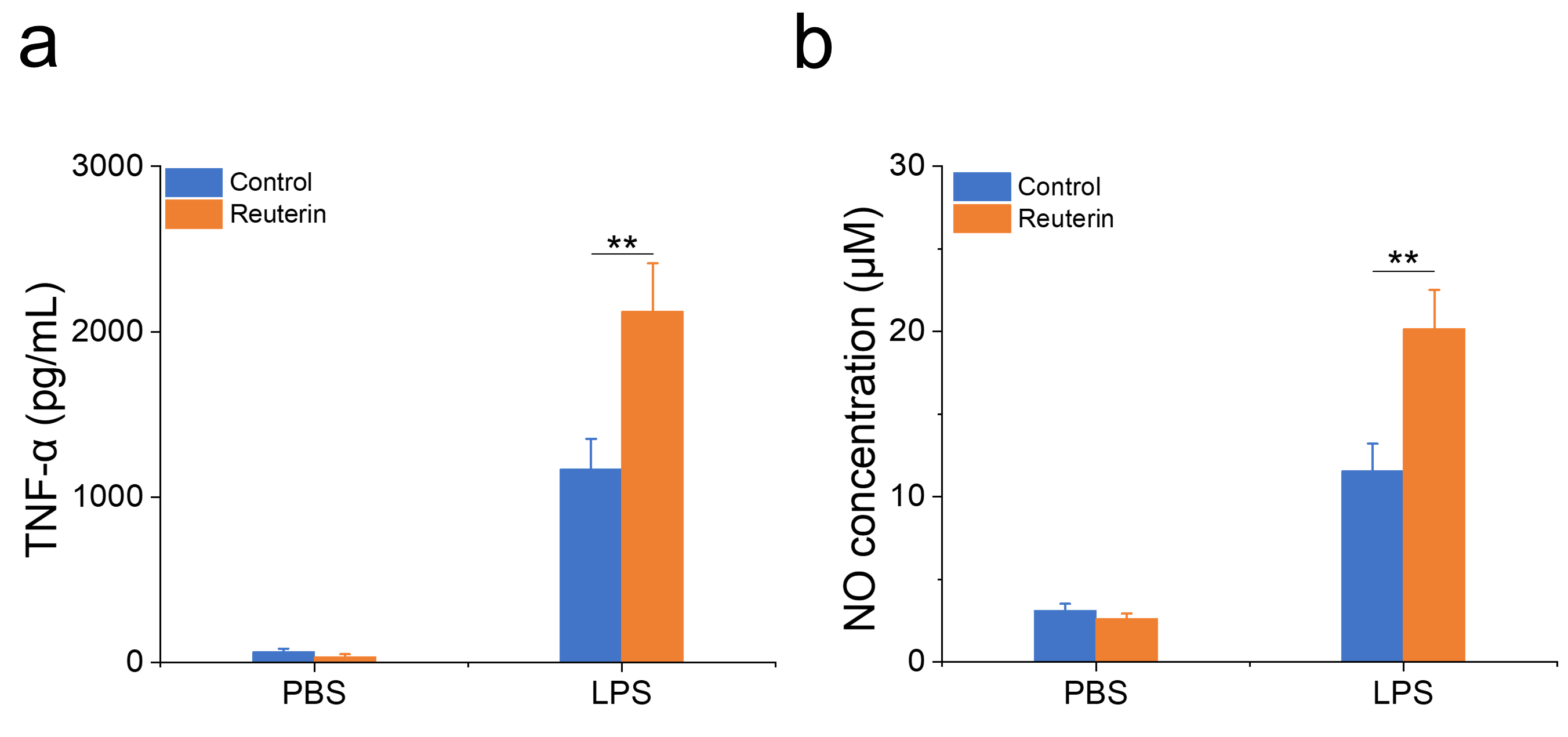
**

**Figure S5.** Reuterin induces trained immunity in bone marrow-derived macrophages. a, b) TNF-α and NO levels in bone marrow-derived macrophages treated with reuterin (10 μM) or PBS, followed by 100 ng/mL LPS stimulation after a 5-day resting period (n = 3). Data represent means ± SD from 3 independent experiments. ***p* < 0.01.

**
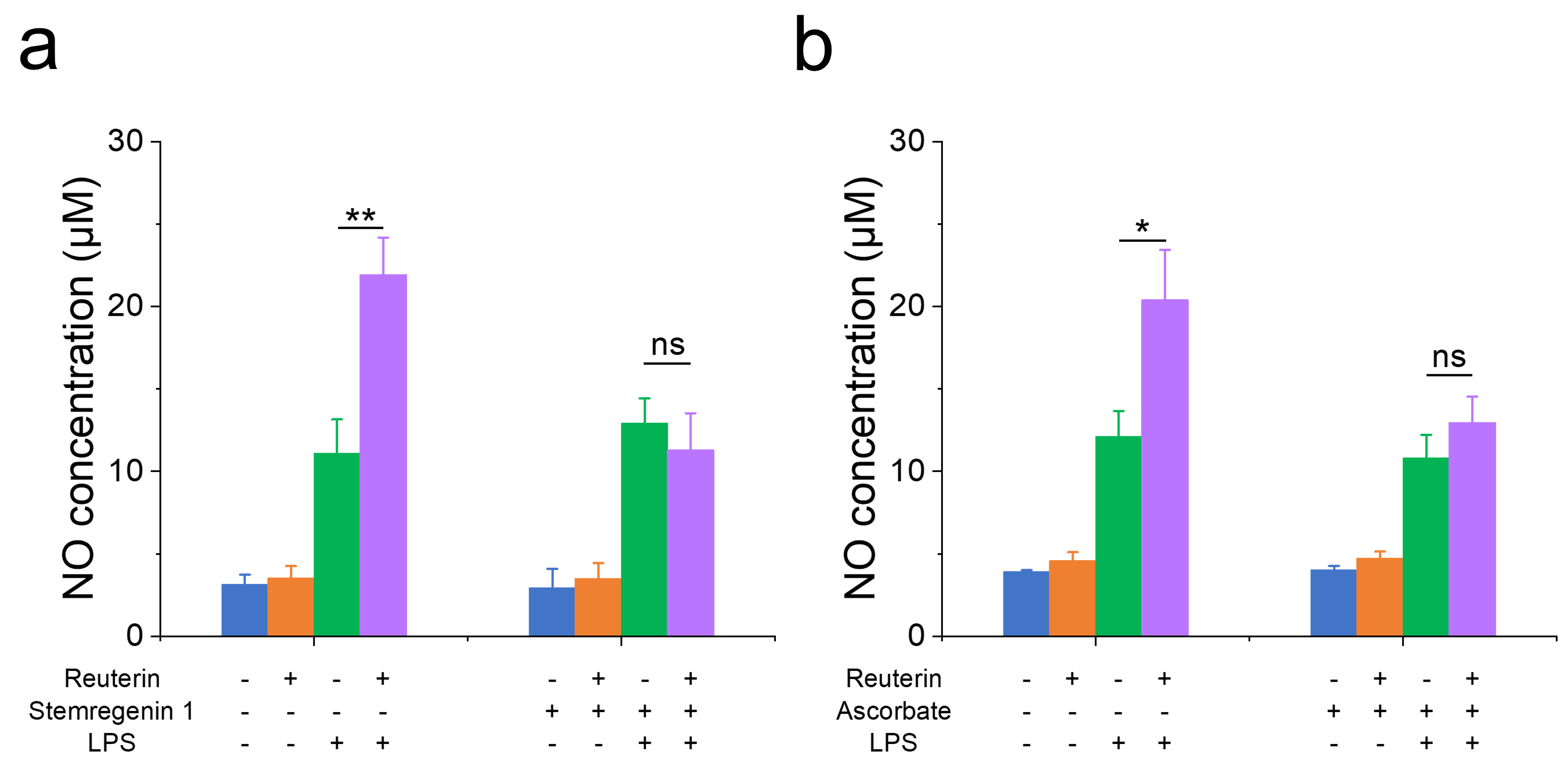
**

**Figure S6.** Reuterin induces trained immunity through the AHR-ROS-HIF1α signaling pathway. a) NO levels in macrophages pre-treated with the AHR inhibitor stemregenin-1 (10 μM), trained with reuterin, rested for 5 days, and stimulated with LPS (100 ng/mL) for 24 hours (n = 3). b) NO levels in macrophages pre-treated with the HIF-1α inhibitor ascorbate (25 μM), trained with reuterin, rested for 5 days, and stimulated with LPS (100 ng/mL) for 24 hours (n = 3). Data represent means ± SD from 3 independent experiments. ns, not significant; **p* < 0.05, ***p* < 0.01

**
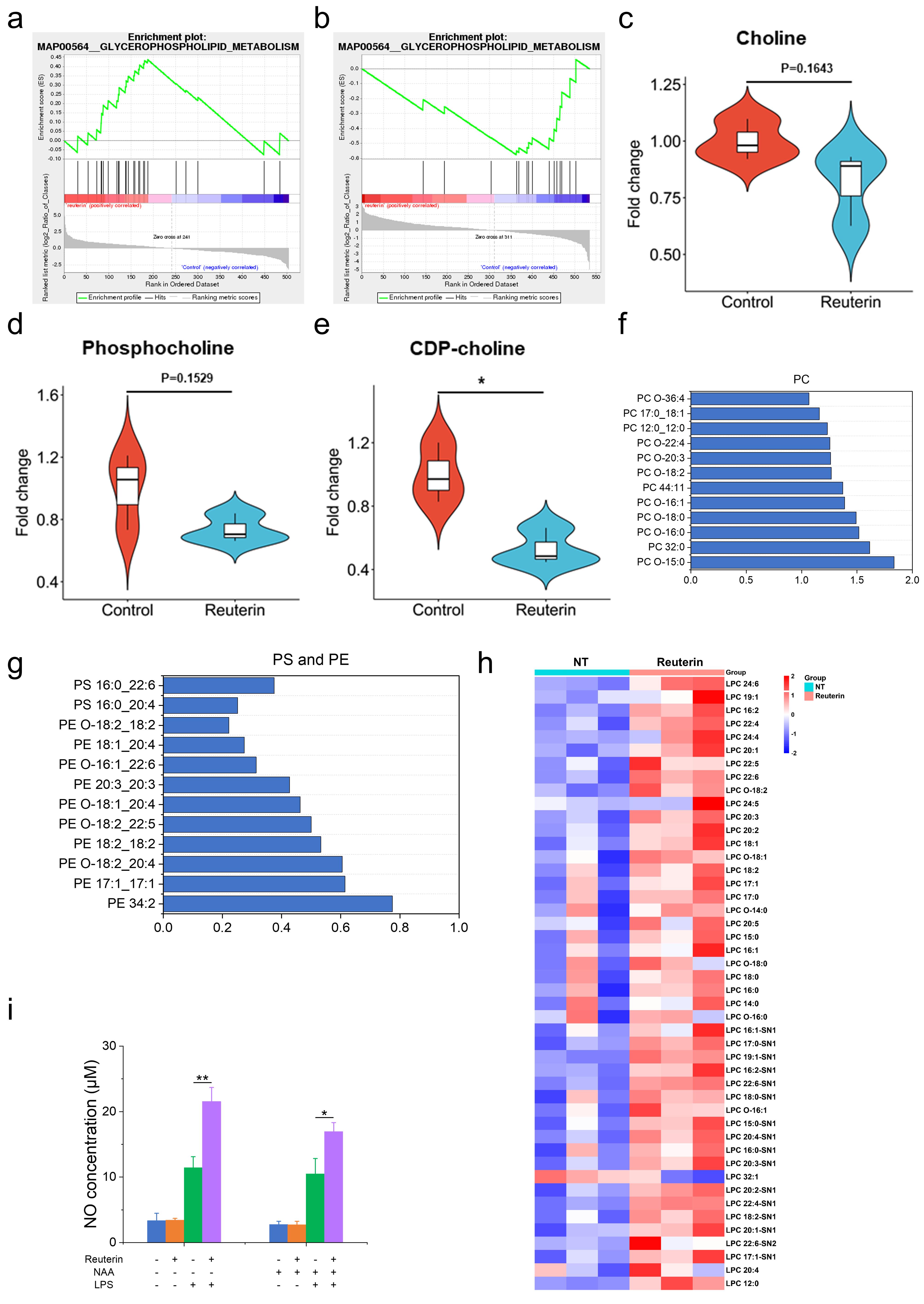
**

**Figure S7.** Reuterin-induced trained immunity is driven by glycerophospholipid metabolism. a, b) GSEA enrichment plot of glycerophospholipid metabolic pathways in negative (a) and positive (b) ion mode. c, d, e) Relative fold changes in the levels of choline (c), phosphatidylcholine (d), and CDP-choline (e) among metabolites. f, g) Relative fold changes in the levels of phosphatidylcholine (PC; f), phosphatidylserine (PS), and phosphatidylethanolamine (PE; g) among metabolites. h) Heatmap depicting the Lysophosphatidylcholine (LPC) changes in macrophages following control and reuterin treatment. i) NO levels in macrophages pretreated with the PLA2 inhibitor 1-naphthylacetic acid (NAA; 50 μM), trained with reuterin , rested for 5 days, and stimulated with LPS (n=3). **p* < 0.05, ***p* < 0.01.


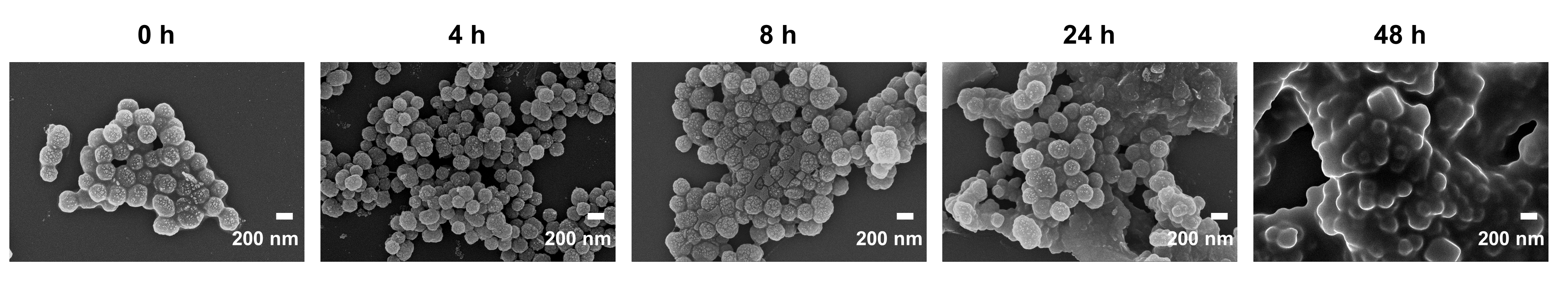


**Figure S8.** Morphological changes of COF-Reuterin under a simulated acidic tumor microenvironment were examined by scanning electron microscopy (SEM) at multiple time points. Samples were collected at 0, 4, 8, 24, and 48 hours to evaluate time-dependent structural alterations.

**
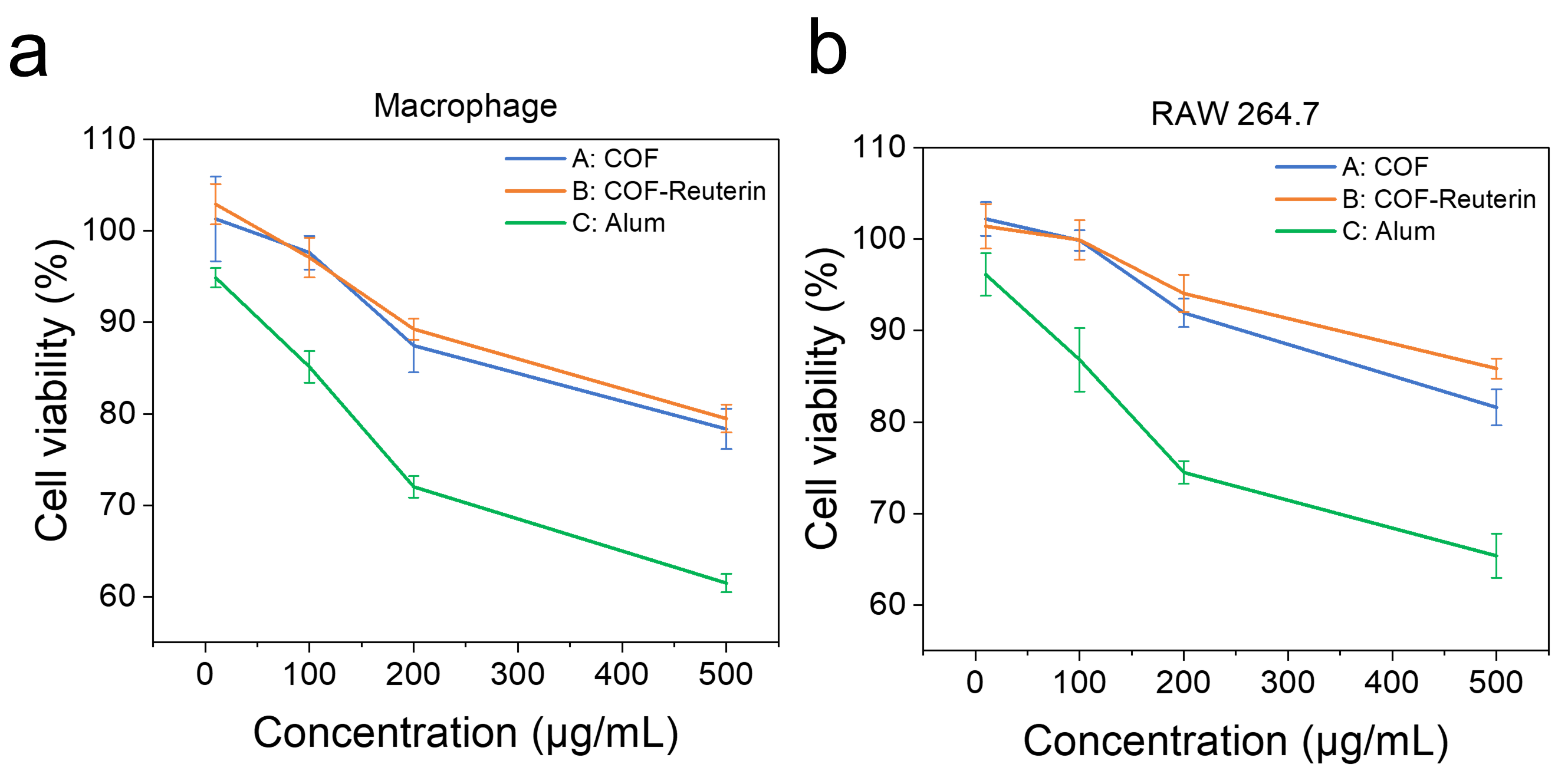
**

**Figure S9.** In vitro studies of COF-Reuterin. a, b) Cell viability of macrophages (a) and RAW 264.7 (b) cells following 24-hour incubation with COF, COF-Reuterin, and Alum was assessed using the CCK-8 assay (n = 3). Data represent means ± SD from 3 independent experiments.


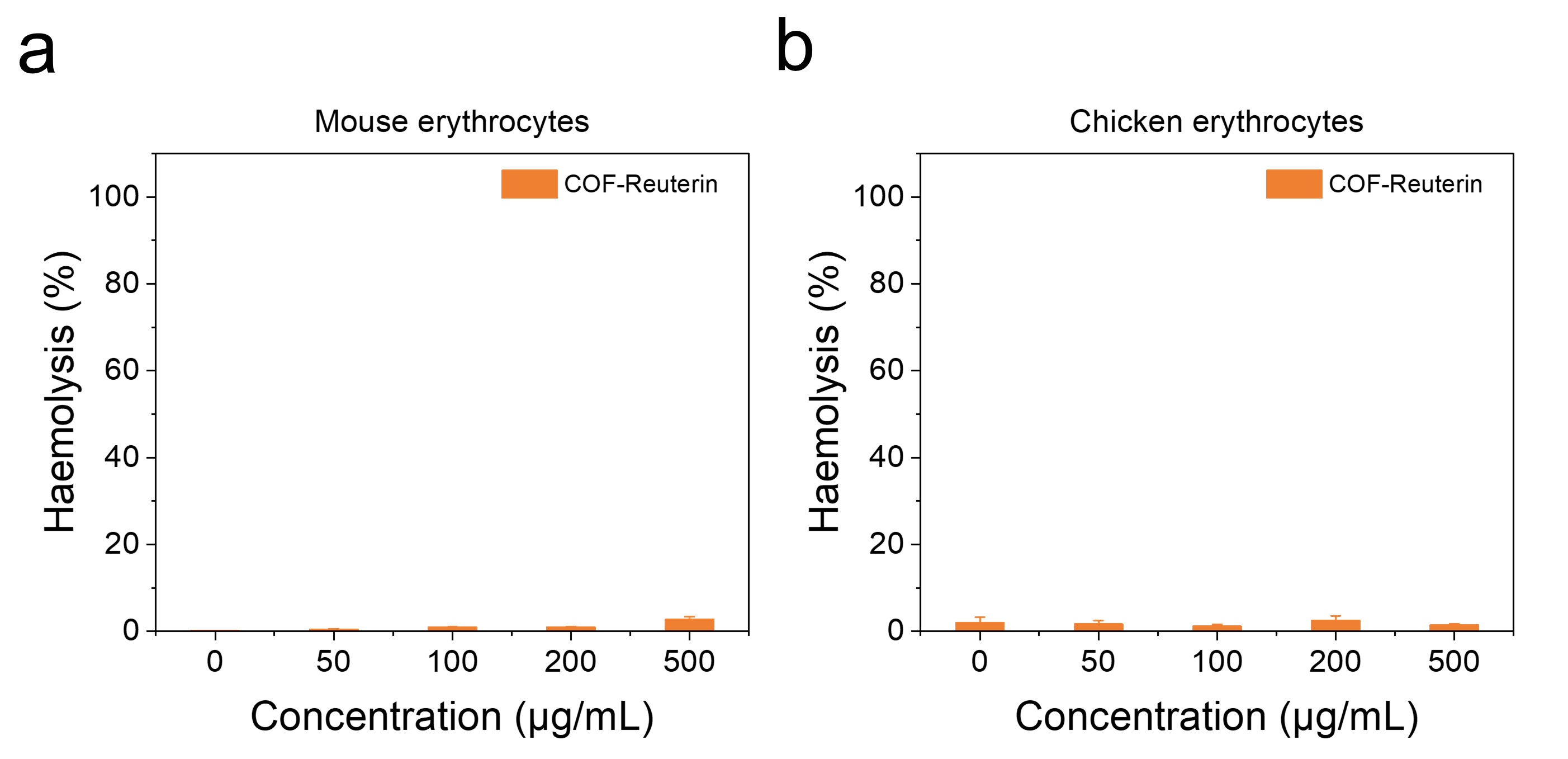


**Figure S10.** In vitro assessment of the hemolytic activity of COF-Reuterin on mouse (a) and chicken (b) erythrocytes (n = 3). Data represent means ± SD from 3 independent experiments.

**
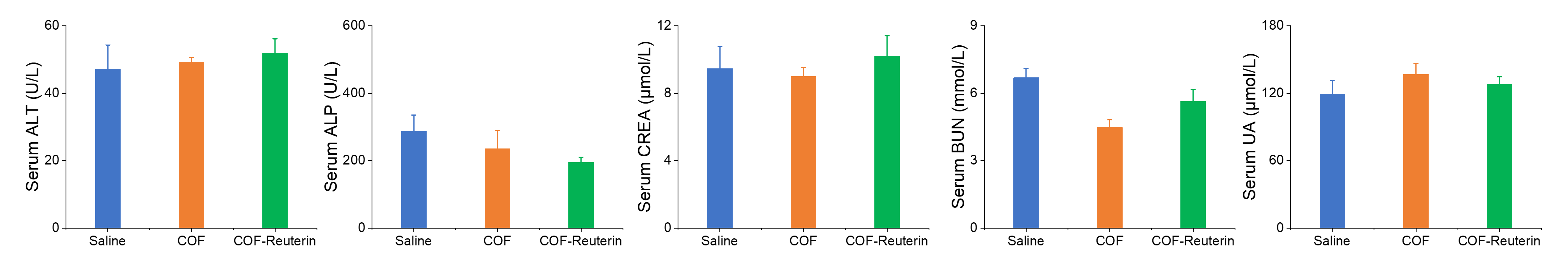
**

**Figure S11.** Serum samples were collected from mice treated with saline, COF, or COF-Reuterin, and the levels of alanine aminotransferase (ALT), alkaline phosphatase (ALP), creatinine (CREA), blood urea nitrogen (BUN), and uric acid (UA) were measured. (n = 3). Data represent means ± SD from 3 independent experiments.

**
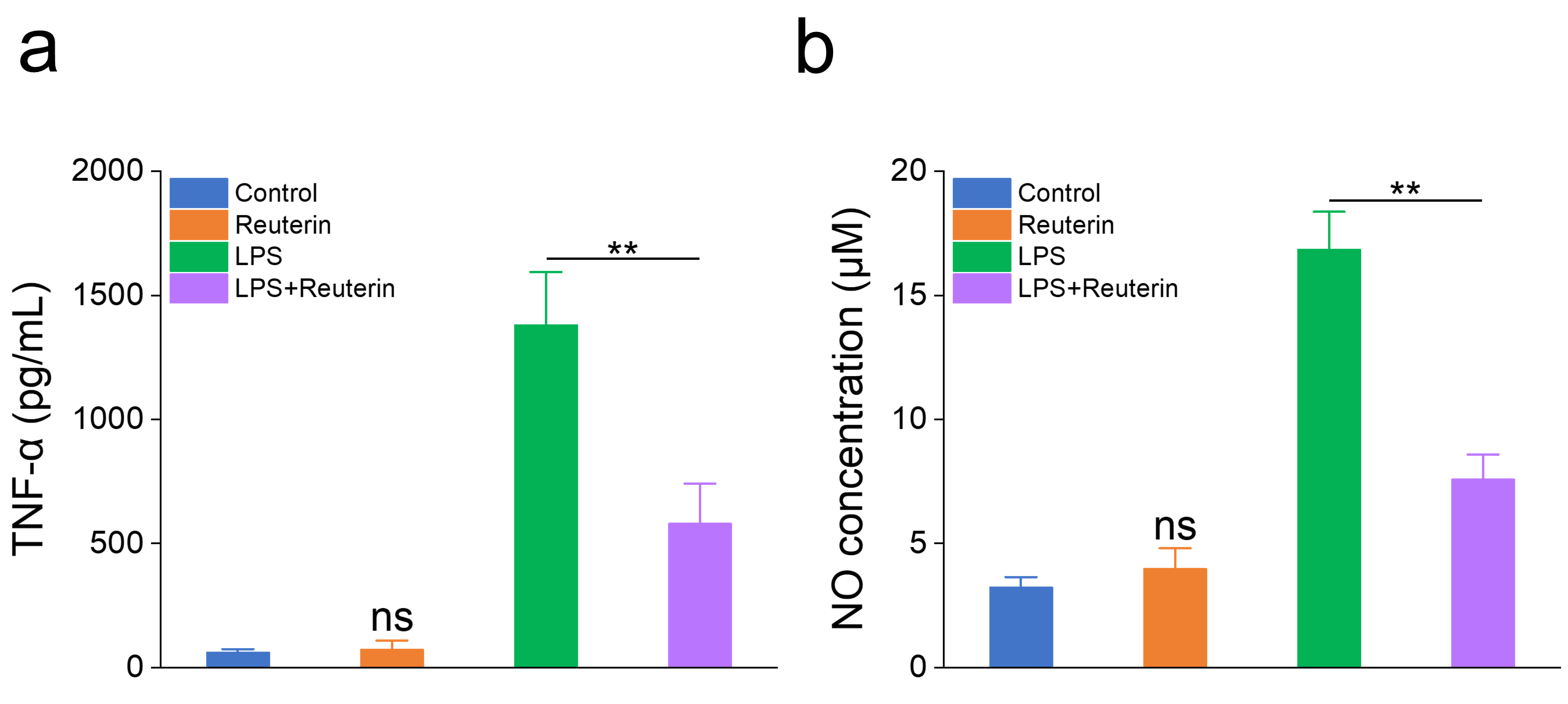
**

**Figure S12.** Reuterin inhibits LPS-induced macrophage inflammatory response. (a) TNF-α levels in macrophage supernatant (n = 3). (b) Nitric oxide levels in macrophage supernatant (n = 3). Data represent means ± SD from 3 independent experiments. **p < 0.01.

**
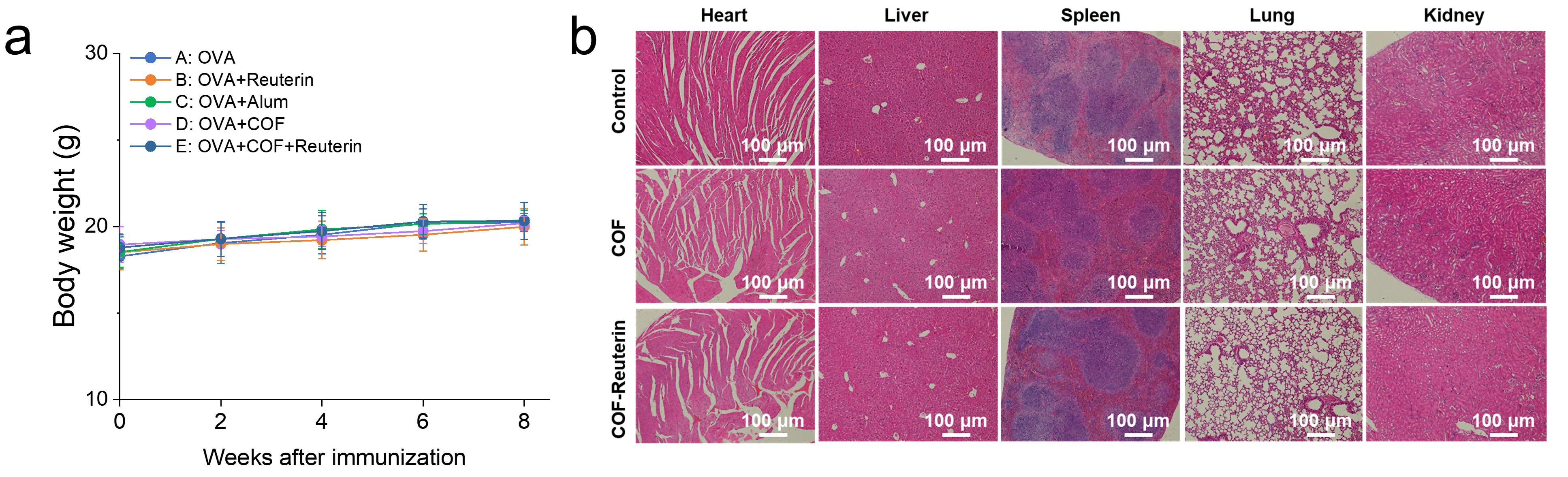
**

**Figure S13.** Safety and tolerability of COF-Reuterin in vaccine studies. a) Body weight. b) Major organs were assessed using hematoxylin and eosin staining assay on week8 following immunization (scale bar, 100 μm). Data are presented as means ± SD.


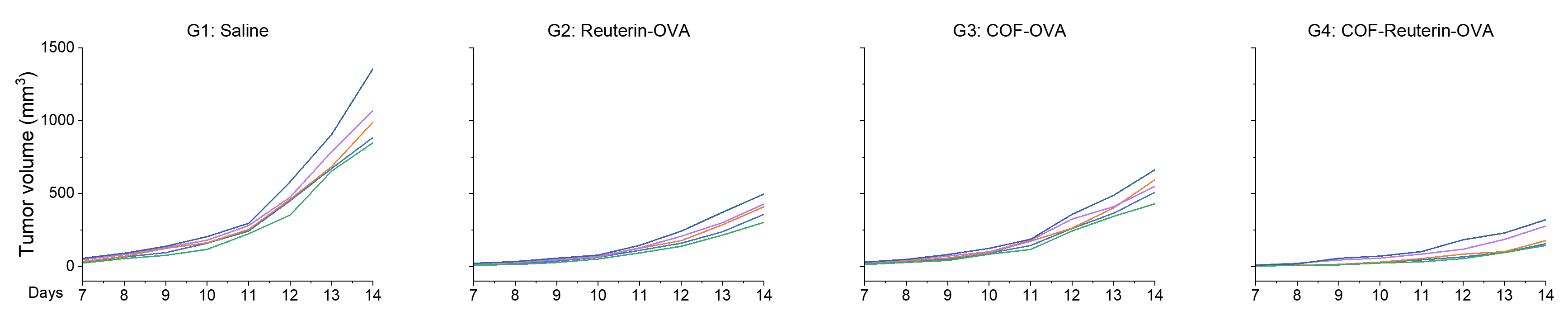


**Figure S14.** Individual tumor growth curves for the prevention of B16-OVA tumors.

**
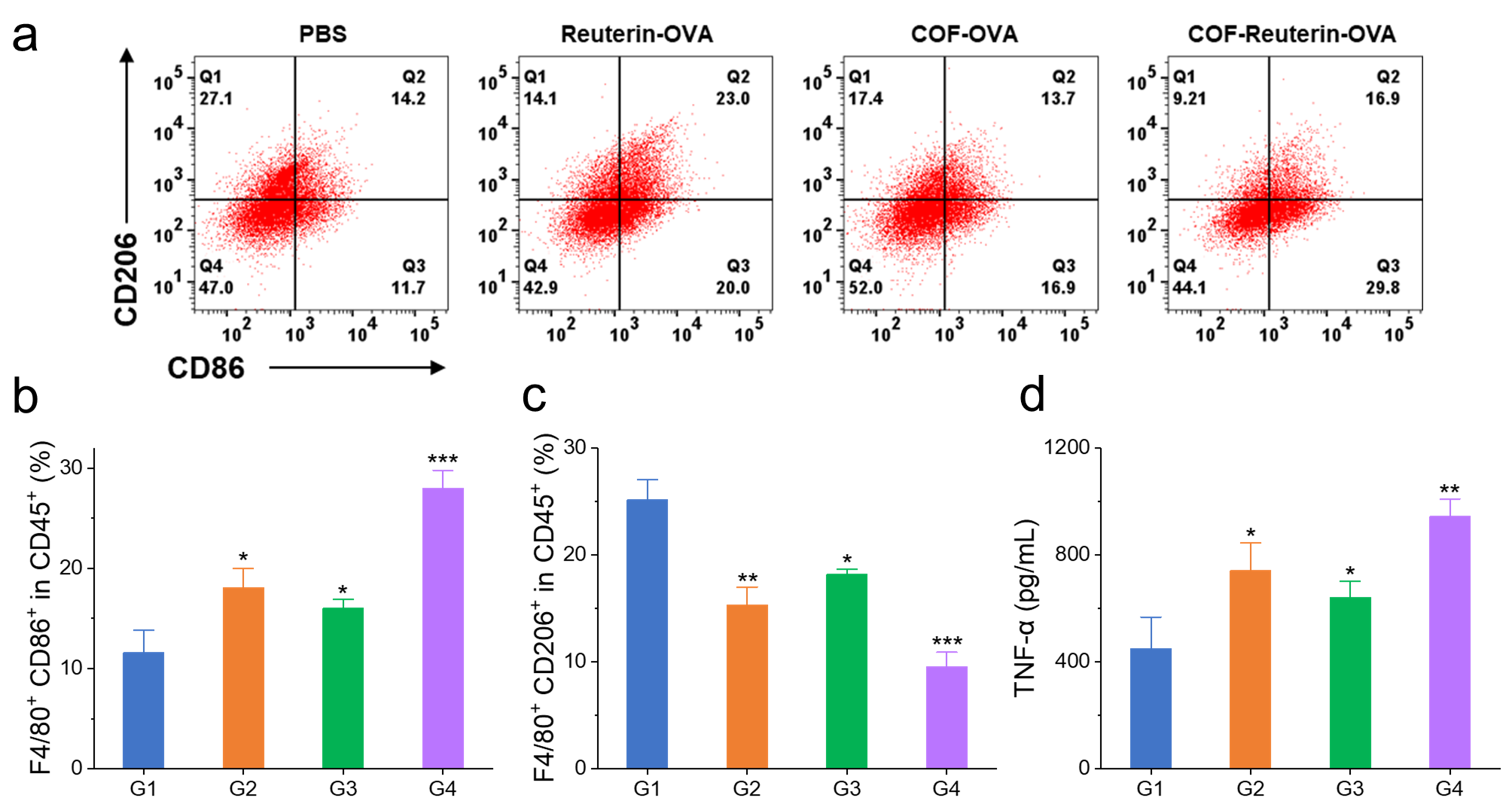
**

**Figure S15.** Covalent Organic Framework-loaded Reuterin induces trained immunity. a) Representative flow cytometry plots showing the proportions of CD86^+^ (M1 macrophages) and CD206^+^ (M2 macrophages) within CD45^+^F4/80^+^ cells isolated from tumor tissues of B16-OVA tumor-bearing mice pretreated with PBS, Reuterin-OVA, COF-OVA, or COF-Reuterin-OVA. (b, c) Quantification of the proportions of F4/80^+^CD86^+^ (b) and F4/80^+^CD206^+^ (c) within CD45^+^ cells from the tumor tissues of B16-OVA tumor-bearing mice (n = 3). d) TAMs were isolated from tumor tissues of B16-OVA tumor-bearing mice pretreated as above and stimulated with LPS. TNF-α levels were subsequently measured (n = 3). Data represent means ± SD from 3 independent experiments


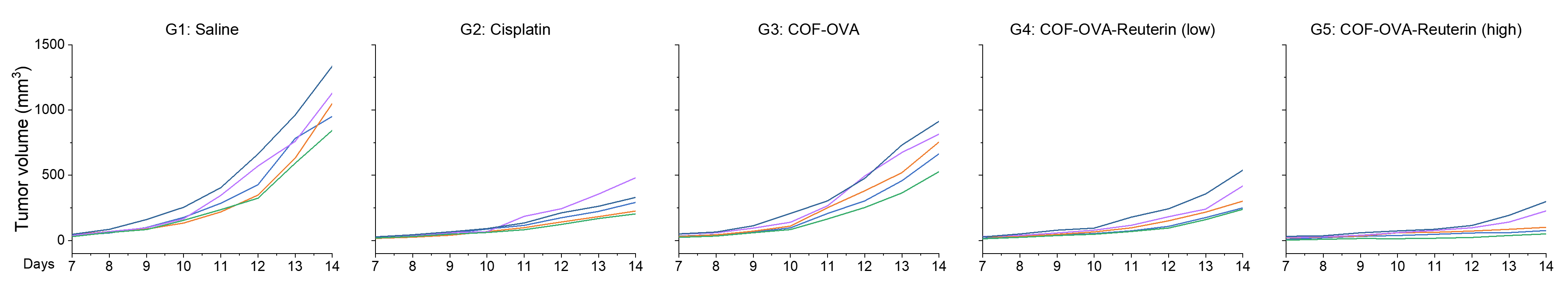


**Figure S16.** Individual tumour growth curves of B16-OVA tumor-bearing mice after subcutaneous injection.


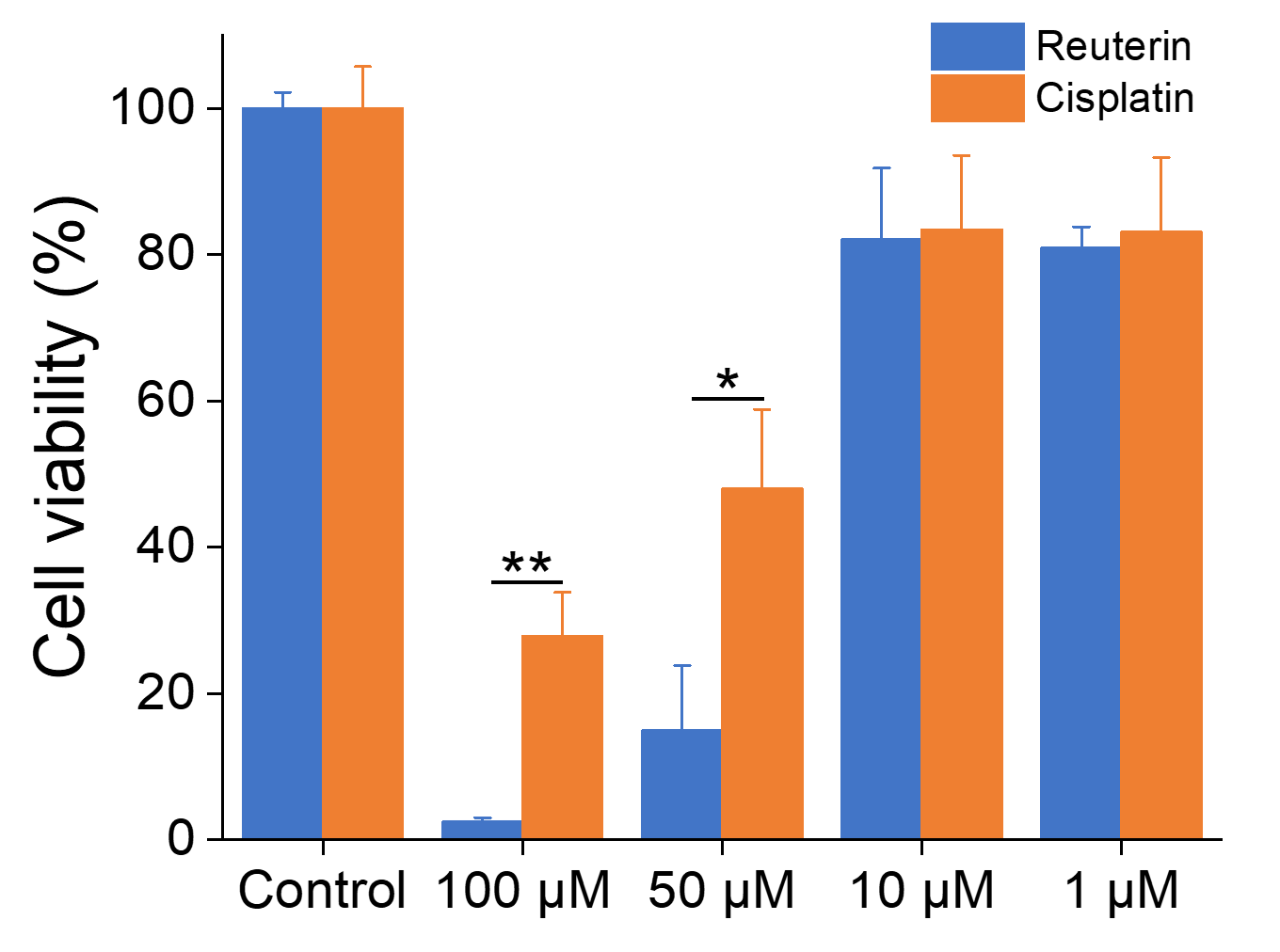


**Figure S17.** Analysis of the cytotoxic effects of reuterin and cisplatin on B16-OVA cells (concentrations ranging from 1 μM to 100 μM) (n = 3). Data represent means ± SD from 3 independent experiments. *p < 0.05.**p < 0.01.

**
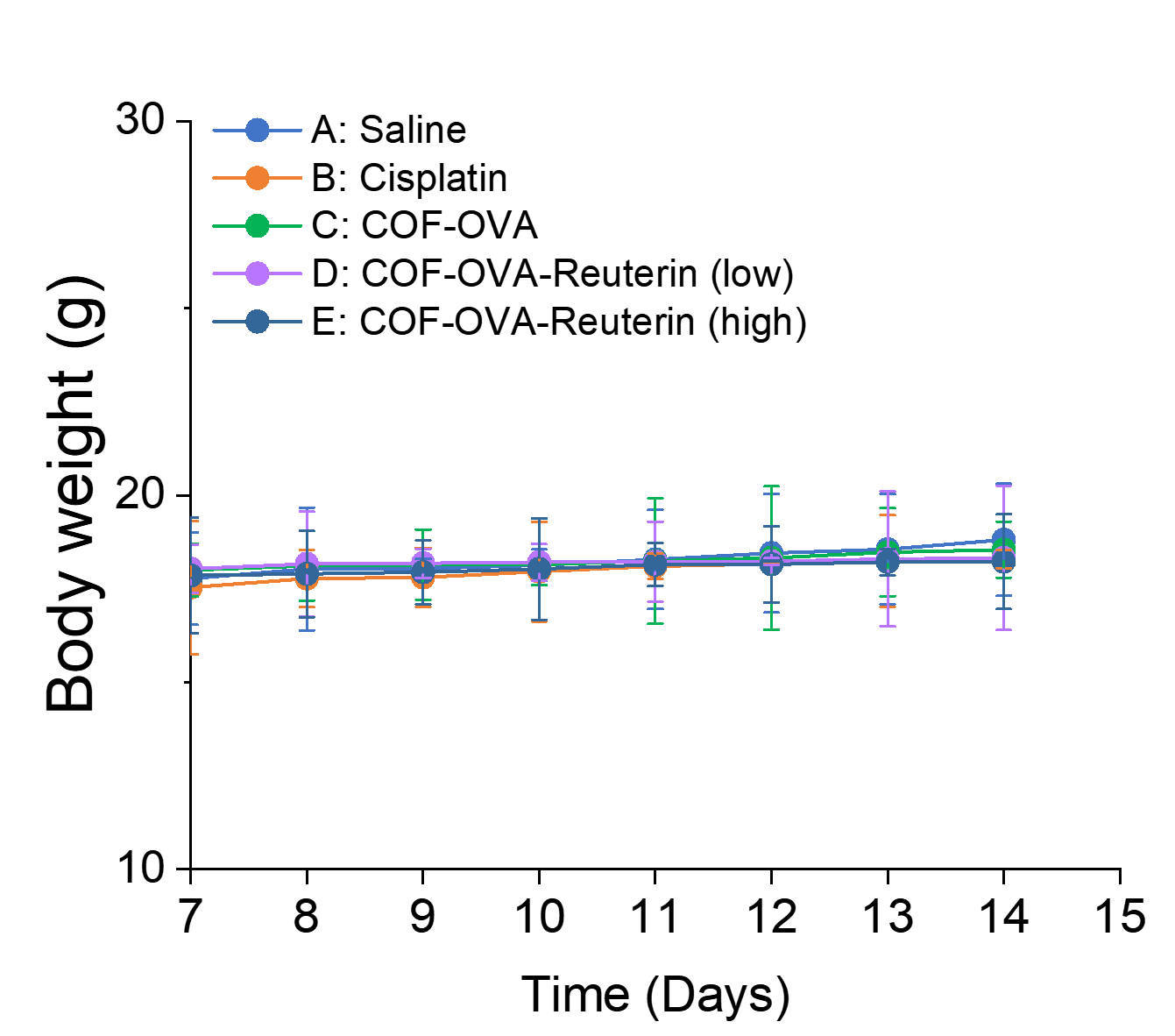
**

**Figure S18.** Body weight of B16-OVA tumor-bearing mice after treatment with COF-OVA-Reuterin.


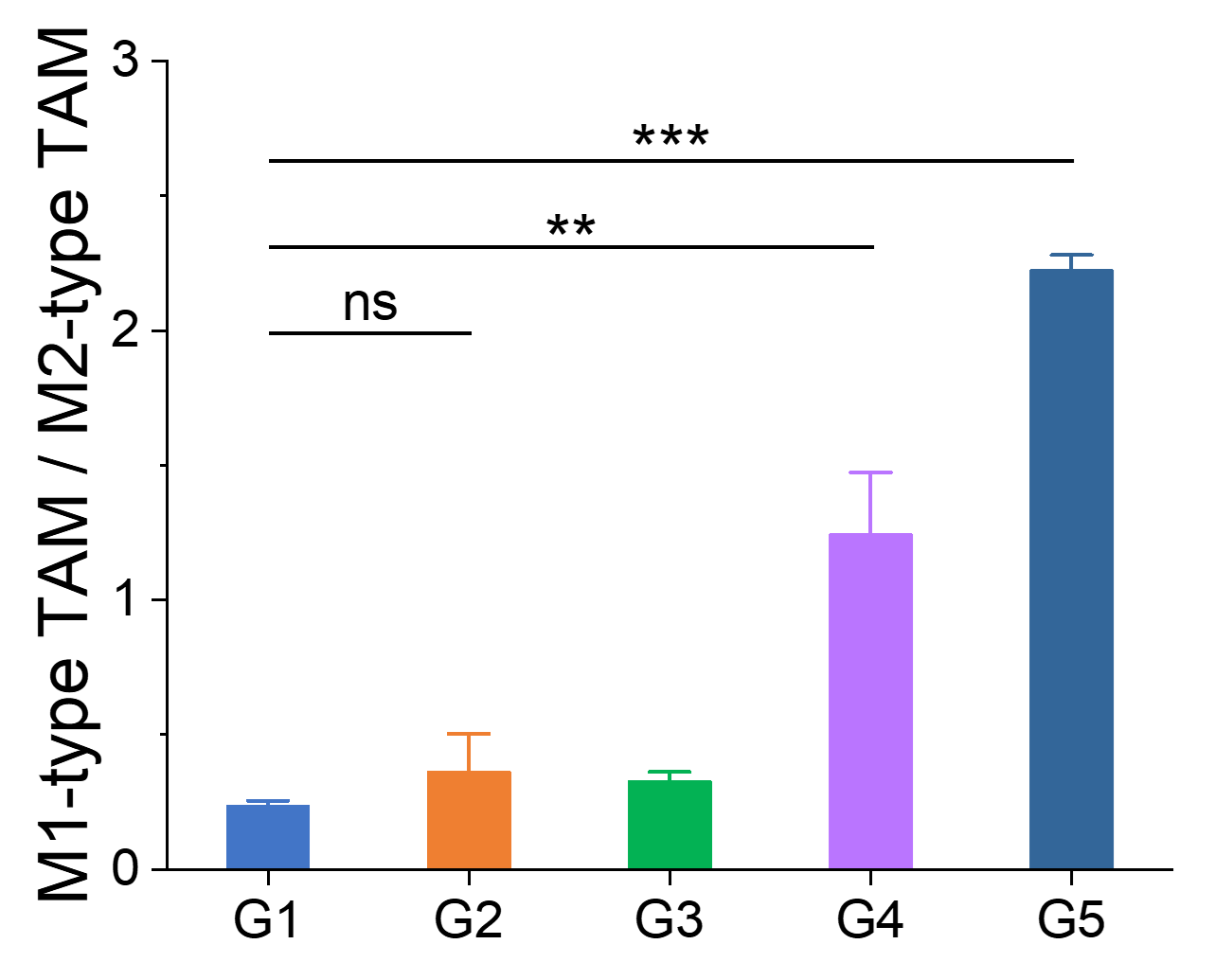


**Figure S19.** Flow cytometry analysis of the M1/M2 macrophage ratio in tumor tissues (n = 3). Data represent means ± SD from 2-3 independent experiments. ns, not significant; ***p* < 0.01, ****p* < 0.001, *****p* < 0.0001.


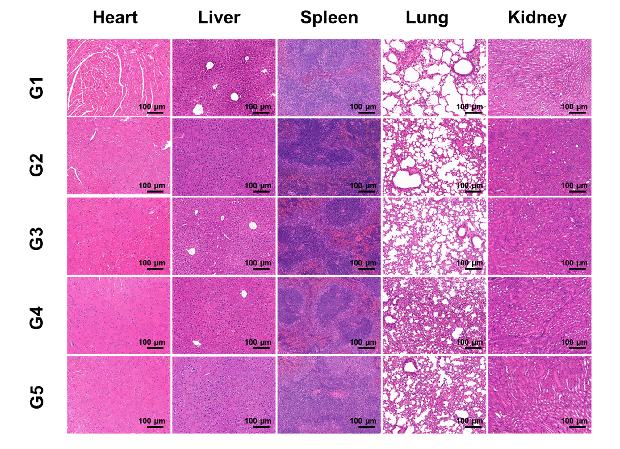


**Figure S20.** Major organs including the liver, kidney, heart, and lung were examined by hematoxylin and eosin (H&E) staining after one month of treatment to assess potential histopathological changes (scale bar, 100 μm).


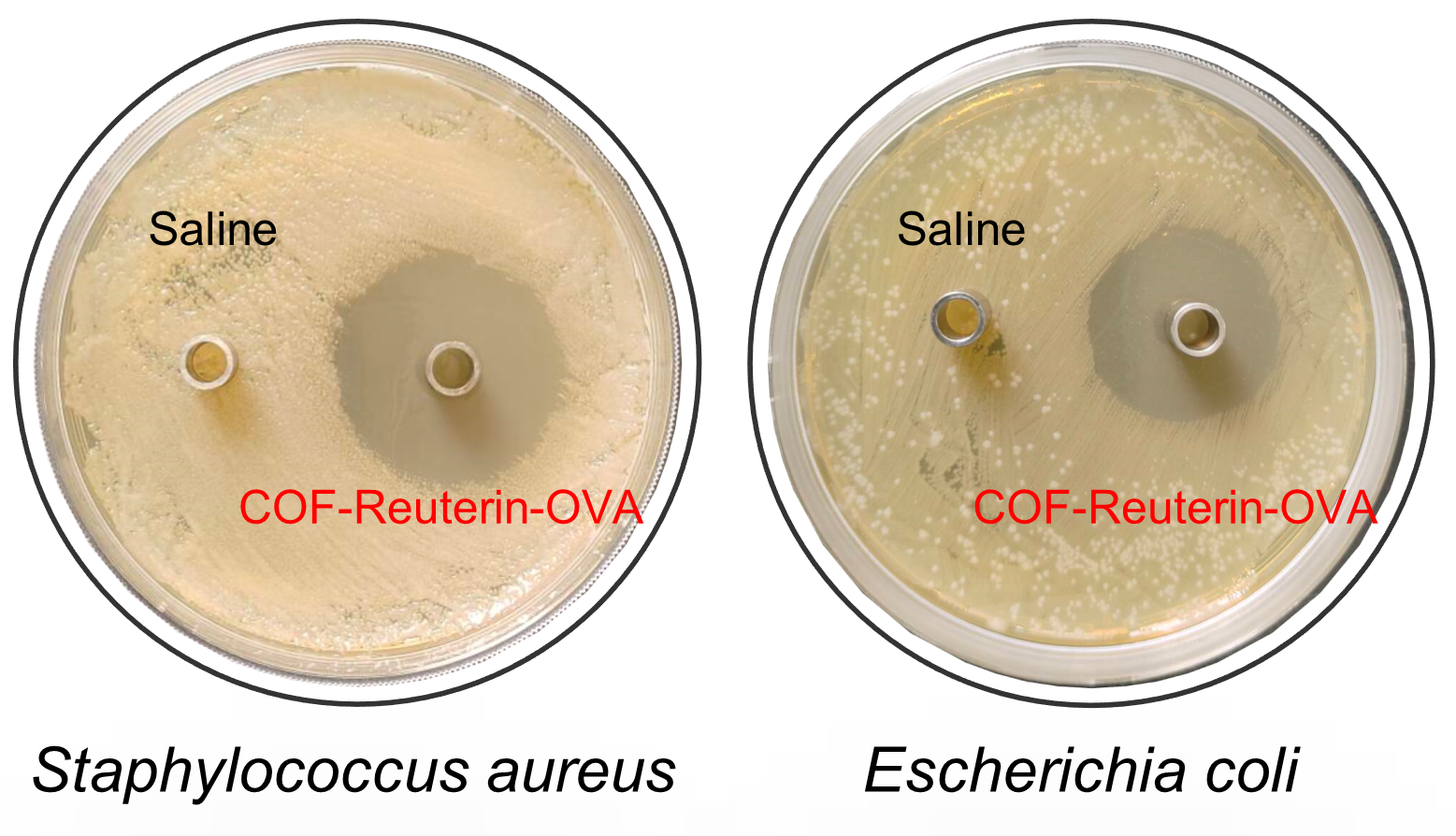


**Figure S21.** Effects of COF-Reuterin on intratumoral bacteria. Oxford cup assay assessing the antimicrobial activity of COF-Reuterin against *Staphylococcus aureus* and *Escherichia coli*.


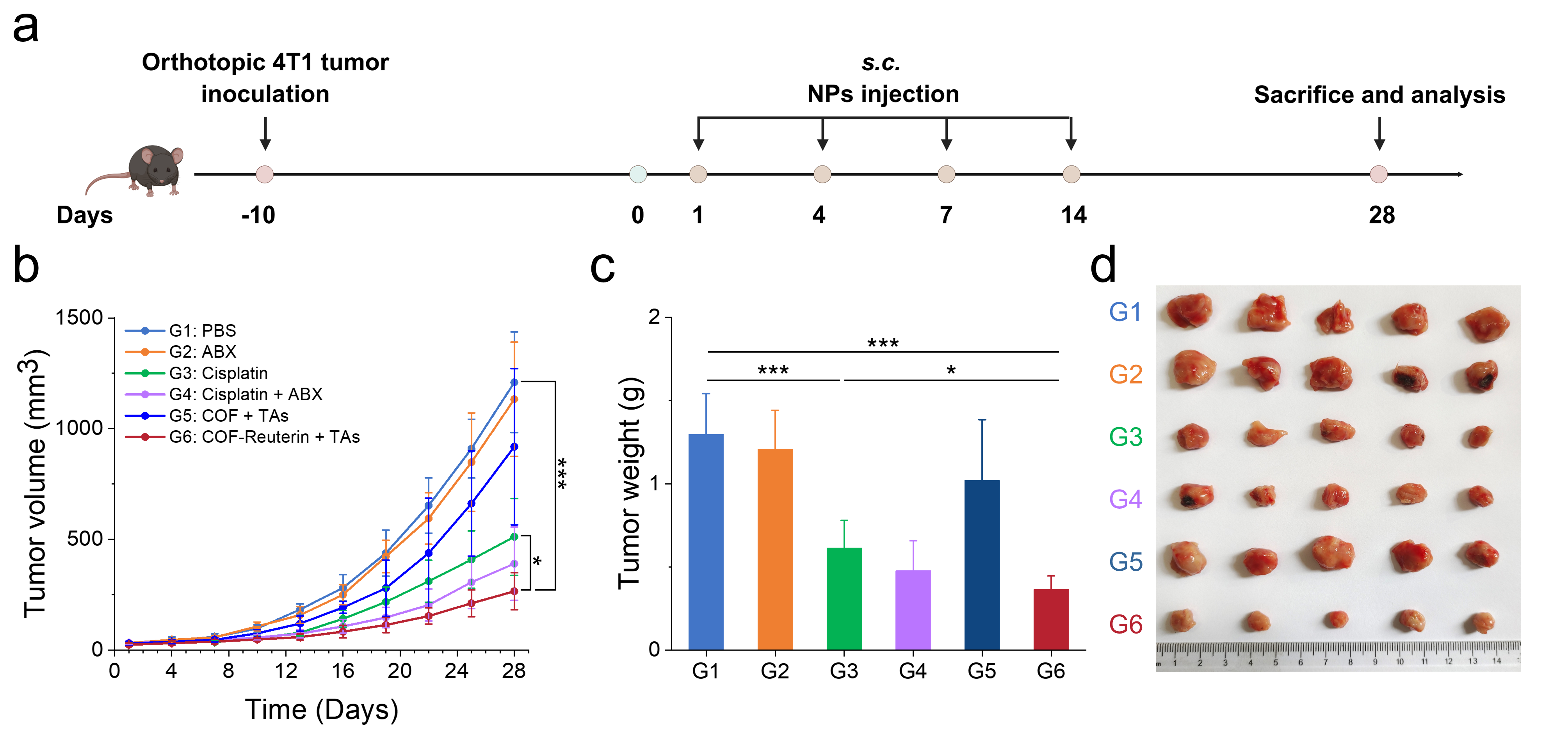


**Figure S22.** COF-Reuterin vaccine significantly improves anti-tumor immune responses in the 4T1 breast cancer model. a) Schematic representation of the experimental timeline for anticancer studies in the 4T1 model. b) Tumor growth curves of 4T1 breast cancer model mice treated with different formulations (n = 5). c) Quantification of tumor tissue weights from each group (n = 5). d) Representative images of tumor tissues from treated mice (n = 5). TAs: tumor antigens derived from 4T1 tumor cell membranes.

**Tabel S1.** Primer sequences in qRT-PCR analysis.

| **Gene** | **Forward (5’ – 3’)** | **Reverse (5’** – **3’)** |
| --- | --- | --- |
| TNF-α | GACGTGGAACTGGCAGAAGAG | TTGGTGGTTTGTGAGTGTGAG |
| IL-6 | CCAAGAGGTGAGTGCTTCCC | CTGTTGTTCAGACTCTCTCCCT |
| IL-1β | GCAACTGTTCCTGAACTCAACT | ATCTTTTGGGGTCCGTCAACT |
| Chka | GGGTGGTCTCAGTAACATGCT | GAACCCTGGACTCACCATCTT |
| Chkb | AGGATGCTAAGTGCCCAGAG | TCACGGGACAAACGCTCAG |
| Pcyt1a | GATGCACAGAGTTCAGCTAAAGT | TGGCTGCCGTAAACCAACTG |
| Chpt1 | ACTGAGATCCAGGTAGCTTTAGT | GTAGACCCATTCTTGCCAACA |
| HIF-1α | ACCTTCATCGGAAACTCCAAAG | CTGTTAGGCTGGGAAAAGTTAGG |
| β-actin | GGCTGTATTCCCCTCCATCG | CCAGTTGGTAACAATGCCATGT |
